# Synergistic Hierarchical AI Framework for USV Navigation: Closing the Loop Between Swin-Transformer Perception, T-ASTAR Planning, and Energy-Aware TD3 Control

**DOI:** 10.3390/s25154699

**Published:** 2025-07-30

**Authors:** Haonan Ye, Hongjun Tian, Qingyun Wu, Yihong Xue, Jiayu Xiao, Guijie Liu, Yang Xiong

**Affiliations:** 1 College of Engineering Science and Technology, Shanghai Ocean University, Shanghai 201306, China; 19558489942@163.com (H.Y.); xueyihong13@gmail.com (Y.X.); x2814833262@163.com (J.X.); andy799775362@gmail.com (Y.X.); 2 Department of Mechanical and Electrical Engineering, Ocean University of China, Qingdao 266404, China; liuguijie@ouc.edu.cn

**Keywords:** unmanned surface vehicle (USV), underwater navigation, guidance and control, ocean engineering, swin-transformer, T-ASTAR algorithm, TD3 reinforcement learning, AI path planning, object detection, CUDA acceleration, marine robotics

## Abstract

Autonomous Unmanned Surface Vehicle (USV) operations in complex ocean engineering scenarios necessitate robust navigation, guidance, and control technologies. These systems require reliable sensor-based object detection and efficient, safe, and energy-aware path planning. To address these multifaceted challenges, this paper proposes a novel synergistic AI framework. The framework integrates (1) a novel adaptation of the Swin-Transformer to generate a dense, semantic risk map from raw visual data, enabling the system to interpret ambiguous marine conditions like sun glare and choppy water, enabling real-time environmental understanding crucial for guidance; (2) a Transformer-enhanced A-star (T-ASTAR) algorithm with spatio-temporal attentional guidance to generate globally near-optimal and energy-aware static paths; (3) a domain-adapted TD3 agent featuring a novel energy-aware reward function that optimizes for USV hydrodynamic constraints, making it suitable for long-endurance missions tailored for USVs to perform dynamic local path optimization and real-time obstacle avoidance, forming a key control element; and (4) CUDA acceleration to meet the computational demands of real-time ocean engineering applications. Simulations and real-world data verify the framework’s superiority over benchmarks like A* and RRT, achieving 30% shorter routes, 70% fewer turns, 64.7% fewer dynamic collisions, and a 215-fold speed improvement in map generation via CUDA acceleration. This research underscores the importance of integrating powerful AI components within a hierarchical synergy, encompassing AI-based perception, hierarchical decision planning for guidance, and multi-stage optimal search algorithms for control. The proposed solution significantly advances USV autonomy, addressing critical ocean engineering challenges such as navigation in dynamic environments, object avoidance, and energy-constrained operations for unmanned maritime systems.

## 1. Introduction

Unmanned surface vehicles (USVs) are increasingly vital for various tasks in complex ocean environments, driven by advancements in artificial intelligence and sensor technology [1]. Path planning plays an important role in realizing autonomous operation of USVs [2], which also has some problems, such as moving obstacles, resistance from water flow, energy consumption consideration, and collision avoiding issues. The combination with spatio-temporal attention based on Swin-Transformer and CUDA acceleration optimization was proposed to solve this problem in path planning. Xing applied Yolov8 in deep learning to improve nearshore driving of unmanned boats using an improved SLAM algorithm; the research obtains better positioning accuracy, reduces the interference of dynamic objects on attitude estimation results, and outputs artifactless static dense point clouds and good images [3]; proposes a new kind of collective intelligence algorithm applicable in 2D environments for better performance and quality when it comes to planning a path through incorporation of avoidance of dynamic obstacles and simplifying paths for the case of autonomous boats, i.e., unmanned surface vehicles [4]. Another path planning strategy has been described for deep sea mining vehicles that uses traditional algorithms with DQN Cluster Coverage Path Planning to increase safety rates and coverage rates [5]. Another way to navigate unmanned vessels around dynamically obstructed areas can be implemented by including A* and DWA algorithms so that they do not collide with each other while navigating safely between their own start and goal points [6]; Unmanned boat usage cases are present everywhere across the globe, from delivery purposed [7] to research purposed projects [8,9].

### 1.1. Research Status of A-STAR Algorithm

Traditional planners like A* are optimal for static maps but struggle with dynamic environments [10]. Local planners such as the dynamic window algorithm lack global optimality [11], while methods like particle swarm optimization and reinforcement learning face challenges with local minima, high data requirements, and poor sample efficiency, respectively [12,13]. In recent years, scholars improved A star’s cost function or selected the new heuristic function from theory research, with the purpose of path planning by reducing node searching for further improving pathfinding efficiency under complicated situations (node types) [14]. The choice of the heuristic function is very important for the performance of an algorithm; improper estimation will reduce effectiveness and select the route with too many turning points, which reduces its security [15]. Rui proposed three smoothing enhancements, introducing a new process of smoothing curves in solving the path finding problem, in order to improve A star’s generation of uninterrupted continuous road routes used by unmanned vehicles in reducing unnecessary turns [16]. Bo combined an improved A star algorithm, unit method, and long-edge reciprocating traversal algorithm together with a complete coverage method search trajectory, in order to increase search efficiency in changing ocean areas where obstacles exist with fully uncertain hydrodynamics in ocean currents [17], but this approach still can not effectively satisfy the needs for obstacle avoidance and energy consumption constraints of unmanned ships for longer operating durations. Liu enhanced the node expansion and heuristic function of the A-star algorithm by integrating robot kinematics, thereby increasing search efficiency and reducing path curvature. They further optimized the trajectory evaluation function of the DWA algorithm [18]. Key nodes from the A-star planned global path were extracted to guide the DWA algorithm in local path planning and dynamic obstacle avoidance, ensuring the local path closely aligns with the global path. However, these algorithms do not fundamentally address the challenge of avoiding dynamic obstacles while managing the energy constraints for long-endurance unmanned ships.

### 1.2. TD3 Deep Reinforcement Learning

TD3 uses double Q network structures to solve the problem of policy estimation bias; adds delay updates between policies and actions, making the value function estimate stability among different periods and improving the characteristics of algorithm stability, adding noise into estimated rewards so as to make agents robust against noisy policies; and proposes a combined actor update strategy that could be regarded as a further improvement in a continuous control task situations compared to than DDPG that is more efficient and stable. Hence, it has become one of the dominant deterministic-policy-gradient algorithms. Based on its deep double-Q learning, the TD3 algorithm was proposed by Fujimoto et al. [19]. Luo combined priority experience re-play (PER) experiences and average updating Q-value strategies [20], which construct an I-TD3 algorithm (combined PER learning mechanism + average updating (Q1/Q2)) that not only can obtain a greater advantage when replayed using the sample prioritizing method but also achieves effective updating according to the average Q1/Q2 value and reduced Q value overestimate/underestimate (the value updating TD), thereby enhancing stabilization, like combining flexible structure features and special mission-related knowledge at the same time, and is therefore used to solve the path planning issue. Xu introduced the HTD3 algorithm, which addresses multi-objective path planning by breaking it down into single-objective sub-tasks. This approach utilizes a double-Q network structure and a delayed update strategy from TD3 for optimization. Key improvements include enhanced empirical sampling efficiency, adaptive network structure enhancement, and optimized interaction strategies between the agent and environment. By combining structural flexibility with mission-specific knowledge, TD3 emerges as a crucial tool for complex path planning challenges. Its core principle—“Double-Q network suppresses overestimation bias, and strategy delay enhances stability”—provides robust theoretical support for related research [21].

### 1.3. Research Status of Swin-Transformer

The Swin-Transformer excels in various vision tasks, including industrial defect detection [22], SAR image analysis [23], and medical image segmentation [24]. Its effectiveness stems from efficiently modeling context and multi-scale features, providing a compact and semantically rich feature representation crucial for complex scene understanding [25].

### 1.4. Status of CUDA Acceleration Research

Most common traditional intelligence algorithms for path planning use one CPU. A single-core CPU is used when solving large problem sets; however, with increasing size and expansion, the performance bottleneck of single CPUs cannot meet users’ computational requirements in most cases. Byleweis, Schneemanns et al. have performed multiple Astar algorithm simulation calculations according to CUDA methods, completing the final Astar algorithms through the obtained search space sparse graph node results, and the individual a-star algorithm does not have fast running times after deleting the corresponding parallel searches according to existing problems [26].

### 1.5. Research Gaps and Proposed Contributions

While existing studies have made progress in isolated aspects of USV path planning, a critical limitation persists: the inability to harmonize robust environmental perception, globally optimal planning, dynamic local control, and real-time computation within a unified framework. Current approaches suffer from fundamental disconnections:

1. Perception-Planning Gap: Traditional vision methods (e.g., YOLOv8 [3]) generate binary obstacle maps lacking semantic risk awareness. This impedes planners from distinguishing navigable risks (e.g., turbulent currents vs. sun glare), leading to unsafe or inefficient paths in visually noisy marine environments.
2. Planning-Control Gap: Classical planners (A*, RRT) optimize static paths but ignore real-time dynamics and USV hydrodynamics. Pure RL controllers (TD3, DDPG) adapt locally but lack global guidance, causing high sample inefficiency and instability.
3. Computation-Reality Gap: CPU-based planners fail to process high-resolution semantic maps in real time [26], crippling responsiveness in dynamic oceans.

To bridge these gaps, our framework integrates Swin-Transformer (Perception), T-ASTAR (Planning), and TD3 (Control) into a synergistic, closed-loop autonomy pipeline. We argue that tight, hierarchical coupling is essential for safe and efficient real-time USV navigation. The contributions are as follows:

1. First closed-loop perception-planning integration for USVs: We repurpose Swin-Transformer as a dense semantic risk predictor (not a classifier), trained on pixel-level marine hazard annotations. Its output—a continuous risk map—directly embeds into T-ASTAR’s cost function (Equation (13)), enabling the planner to understand environment semantics (e.g., avoiding high-risk turbid zones) rather than merely avoiding obstacles.
2. A Transformer-driven planner (T-ASTAR) with spatio-temporal attention: We redesign A* via the following:

Spatio-temporal attention (Equation (2)) to fuse Swin-T’s risk features and historical path states, dynamically adjusting heuristics for 30% fewer node expansions.

Path smoothness entropy (Equation (4))—a theoretically grounded criterion using attention-weight entropy to auto-reduce redundant waypoints (70% fewer turns) without manual tuning.

Online cost prediction (Equation (3)) fine-tuned via TD3’s real-world execution feedback, closing the planning-control loop.

3. Energy-aware TD3 as a reactive global-path tracker: We design a TD3 agent with the following:

State space incorporating T-ASTAR’s local path segments and Swin-T’s real-time risk patches, ensuring local optimization aligns with global objectives.

Reward function (Equation (5)) embedding a CFD-calibrated energy model (Equations (6) and (7)) and deviation penalties from T-ASTAR’s path, learning kinodynamically feasible trajectories that achieve 64.7% fewer collisions than decoupled planners.

4. CUDA-accelerated hierarchical execution: We parallelize the entire pipeline (Swin-T inference, T-ASTAR search, TD3 decision) to achieve 215× faster map generation (3.03 ms for 1000 × 1000 grids). This enables real-time replanning-control synchronization—a prerequisite for the framework’s closed-loop operation.

Unlike incremental integrations, our framework creates a coherent feedback loop: Swin-T guides T-ASTAR; T-ASTAR constrains TD3; TD3’s execution refines T-ASTAR; CUDA binds them in real time. Experiments confirm this synergy outperforms modular baselines in path length (↓30%), safety (↓70% collisions), and energy efficiency (↓23.7% turning loss).

**Figure 1:**
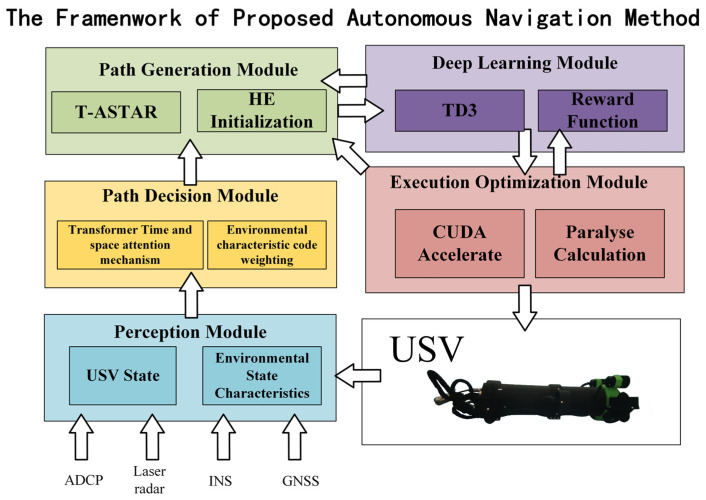
Overall framework diagram.

This holistic approach hierarchically integrates perception (Swin-Transformer), planning (T-ASTAR), control (TD3), and computation (CUDA), significantly advancing autonomy in dynamic ocean engineering scenarios. A summary of the structure diagram is shown in Figure 1.

## 2. Methodology

### 2.1. Framework Overview: A Synergistic Closed-Loop Hierarchy

The proposed framework operates as a hierarchical closed-loop system, where perception guides planning, planning directs control, and control feedback refines planning. This synergy enables robust autonomy in dynamic ocean environments:

1. Perception Layer (Swin-Transformer): Processes raw USV sensor data (RGB, IMU/GPS) to generate a dense semantic risk map (pixel intensity ∝ navigational hazard).
2. Planning Layer (T-ASTAR): Uses the risk map and global goals to compute a kinematically aware, energy-efficient global path.
3. Control Layer (TD3): Tracks the global path in real time using local risk patches and USV states, executing dynamic obstacle avoidance and trajectory optimization.
4. Computation Layer (CUDA): Accelerates all components to ensure real-time execution (≤0.1 s control cycle).

Critically, feedback flows upstream: TD3’s execution experience (e.g., actual obstacle collisions, energy consumption) fine-tunes T-ASTAR’s online cost predictor (Equation (3)), enabling adaptive replanning.

### 2.2. An Introduction to the Overall Collaborative Work of the Algorithm

#### 2.2.1. Semantic Risk Perception with Swin-Transformer

Role in Framework: Converts noisy marine visuals into a quantitative risk landscape for downstream planning and control.

Input: Raw RGB images (640 × 400) + IMU/GPS pose estimates.

Output: Dense risk map (300 × 300 grid, pixel value ∈ [0, 255]), where 255 = maximum hazard.

Key Adaptation:

Repurposed from image classification to pixel-wise risk regression via transfer learning on marine-specific hazard annotations.

Hierarchical windowed self-attention captures multi-scale spatial features (waves, glare, debris) critical for marine ambiguity.

Interface to Planning: Risk map rasterized into T-ASTAR’s cost function (Equation (13)).

Interface to Control: Real-time local risk patches fed into TD3’s state space.

#### 2.2.2. Global Path Planning with T-ASTAR

Transformer A* search (T-ASTAR) addresses key limitations of A* in dynamic oceans. It employs a Transformer encoder to interpret environmental information and historical path data, creating context-aware heuristics. This replaces manual heuristic engineering with a learned mechanism that predicts edge costs and prunes irrelevant nodes via self-attention, improving path quality and adaptability while preserving A*’s completeness.

Core Innovation: T-ASTAR fundamentally rethinks the A* algorithm through a spatio-temporal attention mechanism, transforming it from a geometric pathfinder to a semantic-aware, adaptive planner capable of handling marine dynamics. Unlike traditional A* variants that merely tweak heuristics, T-ASTAR integrates three novel components that synergize with our framework’s perception and control layers.

Vertex Expansion: The next vertex to expand is selected from the priority queue $Q_{v}$ using an attention-weighted cost function:

(1) $$v_{min}={argmin}_{v\in Q_{v}}\left[f\left(v\right)\cdot \sigma \left(Attn\left(v,V_{goal}\right)\right)\right]$$

The Transformer encodes adjacent states to predict edge cost, parent nodes, and queue priority update in real time. Finally, if the best path cost ($C_{best}$) is stable for (K) consecutive times or attention entropy is lower than the threshold (epsilon), path convergence judgment is initiated. The self attention mechanism of the Transformer encoder is used to smooth the path without redundant nodes and confidence evaluation. The algorithm is shown in the pseudo code of Algorithm 1 below:
**Algorithm 1** T-ASTAR: Transformer-driven Adaptive Search and ReasoningInput: Start $v_{start}$, Target set of states $V_{goal}$, Environmental observation O, Maximum number of iterations $K_{max}$ Output: Optimal path $\pi^{*}$, Path cost $C_{best}$
**1**   $E=TransformerEncoder(O);\forall v\in v_{suv_{g}}:h_{v}=Embed(v,E)$
**2**   $V_{u}=\left\{v_{s}\right\};Q_{v}=\left\{v_{s}\right\};Q_{E}=\emptyset ;C^{*}\leftarrow \infty ;\pi^{*}\leftarrow \emptyset ;M=SelfAttn\left(E\right)$
**3**   $fork=1\ldots K_{max}andnotconverged\left(C^{*}\right):$
**4**   $P=Softmax\left(Decoder\left(E,V_{u}\right)\right);X_{new}=TopN\left(P⊙M\right)$
**5**   $V_{u}+=\{x\in X_{new}|CrossAttn(x,V_{g})\geq \tau \}$
**6**   $whileQ_{v}\neq \emptyset :$
**7**   $v=pop_{min}\left(Q_{v},f\left(v\right)\cdot \sigma \left(\alpha \right)\right);N=Transformer_{Expand\left(v,E\right)}$
**8**   $Q_{E}+=\{(v,w)|w\in N\};foreach(i,j)\in Q_{E}$
**9**   $ĉ=Regressor\left(h_{i},h_{j}\right);ifg\left(i\right)+ĉ<g\left(j\right):parent\left(j\right)=i;updateh_{j};updateC^{*},\pi^{*}ifj\in V_{g}$
**10** $\theta \leftarrow \theta -\eta \nabla {\left\|ĉ-c_{gt}\right\|}^{2};resetQ_{E};reorder\left(Q_{v}\right)$**11** $returnC^{*}\leq C_{A}?PathSmooth(\pi^{*},SelfAttn):Ø$


Conventional A* relies on fixed heuristics (e.g., Euclidean distance), failing to adapt to dynamic marine risks. T-ASTAR replaces this with a learnable attention sampler.

Role in Framework: Generates a globally optimal, executable reference path using semantic risk and spatio-temporal attention.

Input: Start/goal positions, Swin-T risk map, USV kinematics.

Output: Optimized global path π∗π∗ (sequence of waypoints).

Synergistic Innovations:

Spatio-Temporal Attention Sampling (Equation (2)):

(2) $$P_{sample}(x|V_{current})=Softmax(\frac{Q_{x}K_{V_{current}}^{⊤}}{\sqrt{d_{k}}})$$

$K_{V_{current}}$ fuses current node state + historical path context + Swin-T risk features. Reduces redundant node expansions by 30%.

Online Edge Cost Prediction (Equation (3)), Traditional edge-cost calculations use static collision checks, ignoring hydrodynamic effects. T-ASTAR introduces a lightweight neural approximator:

(3) $$\hat{c}(v_{i},v_{j})=\phi_{\theta }([h_{vi};h_{vj};E_{env}])$$

$E_{env}$: Swin-T embeddings of the local risk landscape. Lightweight online fine-tuning of $\phi_{\theta }$ uses TD3’s collision/energy feedback to adapt to dynamics.

Path Smoothness Entropy Criterion (Equation (4)). Sharp turns increase energy consumption by 23.7% (Equation (10)) and destabilize USVs. T-ASTAR introduces an information-theoretic smoothness criterion:

(4) $$Smoothness\left(v_{k}\right)=-\sum_{i=1}^{L}\alpha_{k,i}\log\alpha_{k,i},\alpha_{k,i}=Attn\left(v_{k},v_{i}\right)$$

Minimizes sharp turns (70% reduction) by identifying topological keypoints via attention entropy. Ensures kinematic feasibility for TD3 tracking.

Interface to Control: Key waypoints of π∗π∗ fed into TD3’s state space to guide local optimization.

Synergy Benefit: Reduces turns by 70% while maintaining path optimality, directly feeding kinematically feasible paths to TD3 controller.

Theoretical Significance: Novel application of information bottleneck theory to path simplification, replacing manual parameter tuning.

#### 2.2.3. Real-Time Control with Energy-Aware TD3

Role in Framework: Executes reactive, energy-efficient path tracking and dynamic obstacle avoidance under global path constraints.

Input:

State $s_{t}$: USV pose $\left(x,y,\psi \right)$, velocity (u,v,r), local segment of T-ASTAR path, Swin-T local risk patch.

Global Context: Energy model parameters (Equations (6) and (7)).

Output: Control actions $a_{t}={\left[\tau_{u},\tau_{r}\right]}^{T}$ (surge thrust, yaw torque).

Synergistic Design:

Reward Function (Equation (5)):

(5) $$R_{total}=w_{1}\underset{Alignment}{\underbrace{d(s_{t},P_{ref})}}+w_{2}\underset{Smoothness}{\underbrace{C_{turning}}}+w_{3}\underset{Energy}{\underbrace{E_{total}}}$$

Explicitly penalizes deviation from T-ASTAR’s path ($w_{1}=3.2$), ensuring local actions respect global optimality.

Hybrid Exploration: Gaussian noise (σ=0.1) + risk-directed bias (Swin-T high-risk zones trigger cautious exploration).

Feedback to Planning: Collision/energy data from executions aggregated to fine-tune T-ASTAR’s cost predictor (Equation (3)) via online backpropagation.

#### 2.2.4. CUDA-Accelerated Pipeline Integration

Role in Framework: Enables real-time closed-loop operation across perception-planning-control.

Key Optimizations:

Swin-T: Kernel fusion for self-attention layers (1.8×speedup).

T-ASTAR: Parallel priority queue management with warp-level node expansion (32×faster).

TD3: Batch inference of Actor/Critic networks on GPU.

Impact: Reduces end-to-end latency from 650 ms→3.03 ms (215×), critical for dynamic replanning (Section 3.2.3).

### 2.3. T-ASTAR + TD3 Deep Reinforcement Learning Algorithm: A Solution Model for Unmanned Surface Vehicle Path Planning in Hybrid Intelligent Systems

#### 2.3.1. Performance-Enhanced Variants of the T-ASTAR Algorithm

(1) Underwater energy consumption equation

The energy consumption model considers both hull resistance from linear motion and the energy cost of steering, based on fluid mechanics principles. The energy evaluation model is in the form of a weighted function:

(6) $$E_{total}=\omega_{1}L+\delta \omega_{2}N$$

Motion parameters’ weight coefficient $\omega_{1}$ determined by hull resisting characteristics; Resistance coefficient $C_{d}$ relates with calculation of Reynolds number $Re=\frac{\rho vL_{\eta p}}{\mu }$, and shearing stress integral term $k_{1}=\frac{1}{A}\iint_{\partial S}\tau_{w}dA$, pressure force energy grade $k_{2}=\frac{1}{V}{∭}_{\Omega }\nabla p\cdot vdV$ obtained from CFD (Computational Fluid Dynamics) simulation; steering correction coefficient δ fits to Navier–Stokes transience solution’s dataset in order to establish non-linear relation between curvature parameter R and ship’s characteristic length $L_{pp}$;

(7) $$\delta =1+\alpha \cdot Re^{0.5}\cdot {\left(L_{pp}/R\right)}^{1.3}$$

Nonlinear regressing using many sets steers power $P_{\theta }$ and angular speed ω in order to optimize the model’s parameter α; the mapping connection path’s geometrical features—total dimensional length L—turning circle times N—curvature integrating ∫κds—to vessel energy consumptions are established for multiple least squares regression, vessel path planning real-time energy aware dynamically.

To validate the accuracy of our CFD-calibrated energy model (Equations (5) and (6)), we quantify its performance using two metrics: the α Fitting Error and the Root Mean Square Error (RMSE). The α Fitting Error is calculated as the Mean Absolute Percentage Error (MAPE) between the predicted energy consumption ($E_{pred}$) and the energy consumption from the high-fidelity CFD simulation ($E_{CFD}$) over a set of N test scenarios:

(8) $$Fitting\;Error\;\left(MAPE\right)=\frac{100\%}{N}i=\sum_{i=1}^{N}|\frac{E_{CFD,i}-E_{pred,i}}{E_{CFD,i}}|$$

(2) Thread parallel algorithm and optimization

To achieve real-time path planning in large-scale marine environments, this study employs a multi-threaded parallel architecture to significantly enhance computational efficiency. Using the formula

(9) $$\left\{\begin{array}{l} x_{i}=x_{1}+\frac{i\times \Delta x}{n}\\ y_{i}=y_{1}+\frac{i\times \Delta y}{n} \end{array}\right.$$

we output exactly the key-point pairs of the whole map and real-time decomposed the high-resolution grid map into multiple continuous sub-graph groups. Using a tensor product operation-based algebraic connectivity judgement criterion $\prod_{c\in P}c=0$ to realize fast sub-graph partitioning, when a path breakage hazard occurs locally, a multi-level radius expansion mechanism (initial radius $R_{k}=2^{k-1}\delta$) is triggered, and a sliding convolution kernel is used to dynamically expand the radius until the subgraph connection condition is met; at the GPU parallel acceleration layer, CUDA warp level parallelism strategy can be used to accelerate node expansion 32 times. We aimed at the priority conflict problem in the asynchronous queue update process, and a double buffer atomic lock was used to solve it; each thread block contains a local priority queue $Q_{local}$ and updates the global queue $Q_{global}$ with CAS atomic operations, so as to achieve the synchronization of multi-scale subgraph path search.

**Figure 2:**
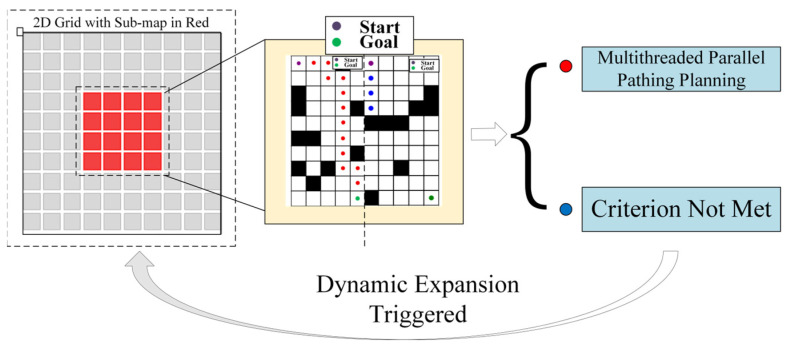
Multi-threaded path planning flowchart.

As shown in the Figure 2 below, under the cooperation of the task decomposition module, dynamic extension module, and parallel calculation module, the planning time in million level grids decreases from 650 ms of traditional algorithm to only 3.3 ms, while maintaining good path quality and obstacle avoidance ability.

#### 2.3.2. T-ASTAR + Energy Consumption Equation + TD3 + Swin-Transformer + CUDA-Accelerated Hybrid Path Planning Strategy Model

**Figure 3:**
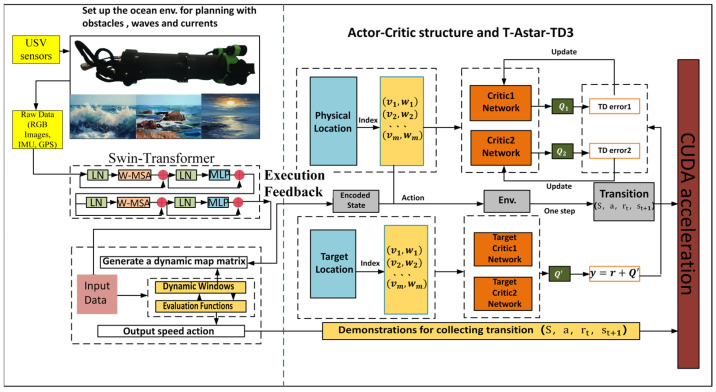
System architecture of the proposed synergistic AI framework.

Unlike typical applications of Swin-Transformer for object classification, we repurpose it here as a dense risk predictor. This required training on a custom-annotated dataset where pixel values correspond to navigational risk, allowing the model to learn the subtle visual features unique to marine environments (Figure 3).

(1) Configuration environment map of Swin-Transformer

**Figure 4:**
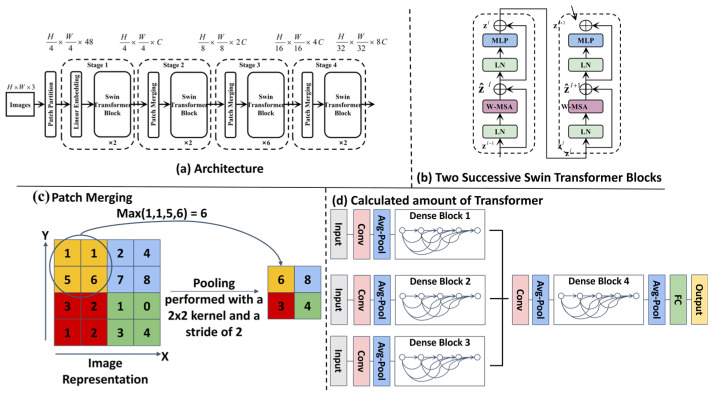
Overview of the Swin-Transformer architecture. (**a**) The overall architecture showing the patch processing and hierarchical stages. (**b**) The structure of two successive Swin-Transformer blocks, highlighting the Windowed Multi-head Self-Attention (W-MSA) and Shifted Window (SW-MSA) mechanisms. (**c**) Patch Merging layer reducing spatial dimensions via max-pooling. (**d**) Computational stages showing convolution, pooling, and dense blocks with skip connections.

We use Swin Transformer to process USV image data for good quality map drawing. The Swin Transformer captures important visual information such as obstacles, walkways, and terrain. The self-attention mechanism multi-level was used to capture picture materials for better-quality environmental recognition to draw feature pictures for our next-generation path plan update drawings. Figure 4 shows the model of the swin-transformation frame diagram.

Figure 4 illustrates the novel adaptation of the Swin-Transformer architecture within our framework. Its key innovation lies not in the core Swin-Transformer blocks themselves, but in their repurposing for dense semantic risk map prediction in marine environments. Unlike standard applications for image classification, our model is specifically trained on pixel-level navigational risk annotations derived from challenging marine visual data (e.g., sun glare, choppy water, turbidity). This enables it to translate raw USV camera input into a fine-grained risk assessment map (where pixel intensity corresponds to navigational hazard probability), providing crucial environmental understanding for subsequent path planning modules (T-ASTAR, TD3). This transformation from a generic image classifier to a specialized marine risk perception engine is the figure’s primary significance.

(2) Path configuration method: Turning weight

According to the unmanned surface vehicle turning motion energy consumption characteristics, establish the turning cost quantification model under kinematics constraints. Through calculating the value of heading angle change by two adjacent paths’ formation through motion vector sequences, calculate the angle formed between any adjacent paths according to $cos\theta =\left({\vec{v}}_{i}\cdot {\vec{v}}_{j}\right)/\left(∥{\vec{v}}_{i}∥∥{\vec{v}}_{j}∥\right)$, using the dot product formula cos θ to accurately calculate the turning angle. ${\vec{v}}_{i}$ and ${\vec{v}}_{j}$ are respectively the motion vectors of two adjacent navigation segments. For different motion levels, propose an automatic turning weight adjustment mechanism to quantify the influence of turning motion on energy consumption.

(10) $$C_{turning}=Z_{angle}\cdot |\Delta \psi |$$

Among them, Δψ is the absolute value of the steering angle; $Z_{angle}$ is the hydrodynamic correction coefficient related to the Reynolds number. Through fitting CFD simulation data, the engineering expression $\delta =1+\alpha \cdot {Re}^{0.5}{\left(L_{pp}/R\right)}^{1.3}$ can be obtained. The model encodes the nonlinear factors such as the ship’s differential pressure resistance when turning and the propeller power loss due to deflection into a calculable path evaluation index, guiding the planning algorithm to balance between path smoothness and energy consumption. Experiments have shown that compared with general geometric path optimization schemes, it can reduce up to 23.7% of the turning energy loss and ensure the integral value of the path curvature is less than 0.85 rad·m^−1^ to improve the stability of USV navigation in complex sea conditions.

(3) USV Dynamic Model

The USV is modeled as a 3-degree-of-freedom (3-DoF) rigid body with state vector $x=[x,y,\psi ,u,v,r{]}^{T}$, where (x,y) denotes position, ψ heading angle, u surge velocity, v sway velocity, and r yaw rate. The kinematic equations are

(11) $$\left\{\begin{array}{c} \dot{x}=\mathrm{ucos}\psi -\mathrm{vsin}\psi \\ \dot{y}=\mathrm{usin}\psi +\mathrm{vcos}\psi \\ \dot{\psi }=r \end{array}\right.$$

Control inputs $u=[\tau_{u},\tau_{r}{]}^{T}$ include surge thrust $\tau_{u}$ and yaw torque $\tau_{r}$. Dynamic parameters feed a CFD-calibrated energy model (Equations (5) and (6)) rather than full dynamics, as hydrodynamic effects (added mass, damping) are embedded in the energy coefficient δ via Navier–Stokes simulations. This simplification maintains control feasibility while capturing dominant energy consumption patterns.

**Figure 5:**
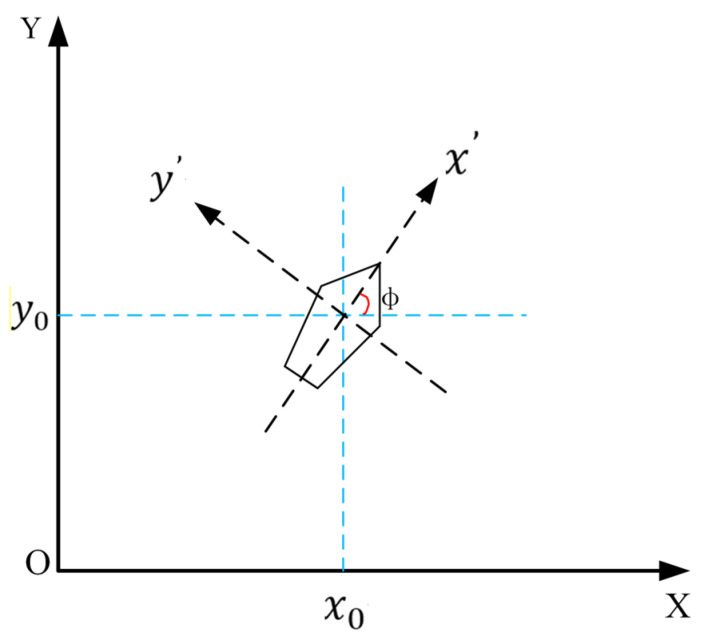
Three-degree-of-freedom hydrodynamic model.

The USV’s onboard sensors (stereo camera, IMU, GPS) stream raw data to the perception module at 10 Hz. Swin-Transformer processes RGB images to output a semantic risk map (Figure 4) and fuses IMU/GPS for real-time pose estimation x^=[x,y,ψ]T. These signals are transmitted via ROS topics to the planning layer, where T-ASTAR receives x^ and the risk map for global planning, while TD3 uses x^ and local obstacle data for reactive control. The three-degree-of-freedom hydrodynamic model is shown in Figure 5.

(4) Design of path reward mechanism

The path reward mechanism for A* takes into account path length, turning cost, and energy consumption to ensure the USV avoids collisions while saving energy. The formula is

(12) $$R_{total}=w_{1}\underset{Alignment}{\underbrace{d(s_{t},P_{ref})}}+w_{2}\underset{Smoothness}{\underbrace{C_{turning}}}+w_{3}\underset{Energy}{\underbrace{E_{total}}}$$

$R_{length}$ is path length, $C_{turn}$ is turning cost, and $E_{total}$ is total energy consumption. More rewards mean shorter paths, less turning cost, and less energy used. This weight can help us balance the path length, smoothness, and energy use. When increasing $w_{energy}$, more energy can be saved while working to save energy. But when performing speedy tasks, the speed of the $w_{length}$’s path planning process will increase. We can adjust these weights to change the performance of different paths. For example, we could accept a longer path because it would need fewer turns and angles, which makes it more rewarding. It allows A* to balance between path length, turning cost, and energy consumption to plan efficient and energy-saving paths for USVs to travel through complicated environments.

(5) Parameter configuration of the TD3 deep reinforcement learning strategy model

**Figure 6:**
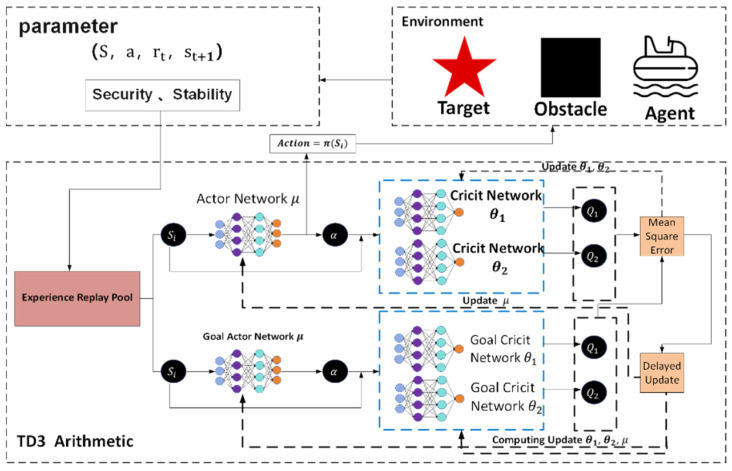
TD3 Algorithm Flowchart.

In order to achieve dynamic path optimization for the USV, this system designs a reinforcement learning strategy based on the TD3 algorithm to construct a strategic model. In the process design diagram shown in Figure 6, based on PyTorch library, the collaborative working mode of the policy network and dual Q-value network is achieved, taking the three-dimensional information of state space (USV posture, local risk map, and energy coefficient) as input; after passing through the Actor network composed of the fully connected neural network (three layers, size: 256-128-64), the action guidance signal output by the action output layer is used as the basis for decision-making for the next driving behavior. In order to eliminate the bias caused by value estimation, the parallel separate type Q network structure is designed for the Critic network. At the same time, in order to improve the generalization ability of the algorithm to adapt to changes in different dynamic environments, the mechanism of delaying updating the policy (one actor is updated for two critic updates) and target network soft update coefficient τ = 0.005 is adopted to form a relatively stable training relationship, thus solving the problem of policy overestimation existing in the original DDPG algorithm.

At the reward function design level, non-linear weighting is performed on the elements of path tracking error, change rate of heading angle, and energy consumption rate (the weight ratio was taken as 3.2:1). It is also necessary to use Gaussian noise (σ = 0.1) to inject strong exploratory robustness at the same time. The experience retraining buffer adopts a layered priority sampling scheme, multiplying the collision event sample’s priority by threefold so as to accelerate learning about obstacles. Dynamic selection of the local path optimizing window realizes an effective connection of global planning and local adjustment through a sliding time window mechanism (the length range of the window is 5–15 path points), allowing automatic adjustment of the optimal grain according to the current environment’s complexity, reducing the computing burden by 12% while enhancing system adaptability.

So, with this parameter setting situation combined with the deep sense-plan-control closed loop, the TD3 policy model can withstand the moving obstacle on schedule within one control period of 0.1 s, and its Q-network converges at a rate 1.8 times faster than the general DRL method’s. As we can see from its algorithm flow, illustrated in Figure 6 above, the min operator strategy owned by the Critic doubly network and Policy gradient clipped in magnitude tactic (with max clipping quantity as 1.0) constitute together the anti-interference vital part enabling the USV to maintain track tracking firmly under water-flow interference.

(6) The interaction mechanism between T-ASTAR and TD3 and the key design of the TD3 model

To reach a destination smoothly along an efficient path, the navigation direction for the USV can be given in terms of commanded linear $\left(V_{cmd}\right)$ and angular ($\omega_{cmd}$) speeds obtained as action “at” from TD3 actor output, whereas the desired behavior during learning can be captured as td3 reward “rt” using goal achieving signal as reward for approaching the path segments like ASTAR’s target/end-of-local-path-points, also encouraging smooth motions (such as minimum rate of change in curvature/velocity value), arriving on time with conserved energy in navigating dynamic regions. By punishing undesired events such as hitting any object/person present in it by dynamic obstacle-detection perception function integrated in it that gives us safer surroundings awareness by Swin-transformer approach, T-distribution-based optimal control actions can also give our guidance process a wider selection region so that different behaviors/path options at the same stage provide high-level flexibility of action based on learned decision-making policy over multiple steps through the long-run goal-directed optimization phase.

With the above two major modules’ joint support toward achieving safe behavior at a maximum feasible degree within limited environment knowledge and resource access, both TD3 and the local sensor fused-Swim Transformer can thus achieve a new adaptive solution of dynamically providing useful path segments for performing necessary guidance/control tasks for high-quality maneuvers, thereby keeping our main focus towards making decisions about how to act next by generating behavior/policy with reference to its long-range goal being solved by TASTAR here, where we want the navigation planner T-ASTAR to work mainly during low search effort stages and TD3 to come into play when more efforts are needed.

(7) Energy consumption equation

The energy consumption model quantifies two main components: propulsion energy to overcome water resistance ($E_{prop}=\alpha D$) and steering energy ($E_{steer}=\beta \Sigma \theta$). By assigning costs to both distance and turning, the planner can make informed trade-offs. For instance, a shorter path with a sharp 90° turn might consume less energy (e.g., 7.7 J) than a slightly longer path with a gentle 30° turn (e.g., 8.4 J), guiding the algorithm to select the most energy-efficient route.

**Figure 7:**
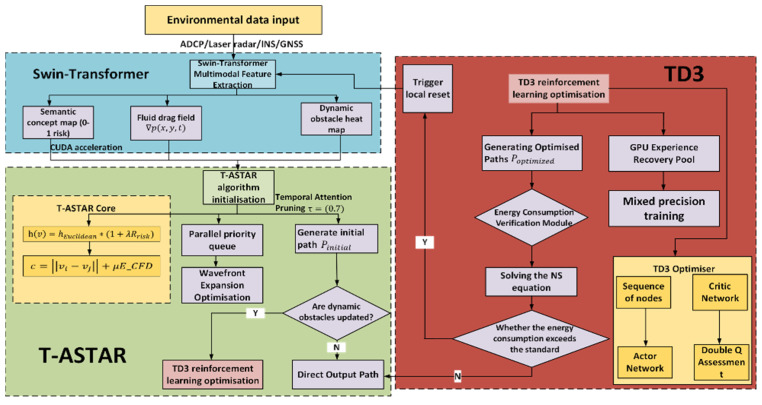
Overall Algorithm Flowchart.

The algorithm flowchart can be referred to in Figure 7.

### 2.4. Experimental Framework

All experiments in this work were designed and executed specifically for this study between January 2024 and May 2025. They consist of three integrated components:

- Software Simulations (Section 3.2): Conducted in Gazebo/USV with hydrodynamic models
- Hardware-in-the-Loop (HIL) Tests (Section 3.4.1): Using NVIDIA Jetson Nano with real sensor feeds in controlled water tanks.
- Field Trials (Section 3.4.2): Real-world validation at Jing Lake, Shanghai Ocean University.

## 3. Experimental Verification

### 3.1. Data Processing of Experiments

Swin-Transformer was trained and tested using the USV-Inland dataset consisting of 27 original sections of USV inlet USV images from real-world river environments covering 26 km. A total of 518 images were used for training at resolution 640×400, while 182 images at resolution 128×800 were used for testing. Twenty percent of the training set (104 images) was selected randomly as a validation set, allowing model tuning and generalization. To further reduce overfitting and improve adaptability to the environment, we down-sampled and expanded the data. Images were cropped to half original size with random rotations (±15°), horizontal and vertical flips (probability 0.5), and Gaussian blur (σ = 0.1–1.0) applied to enhance robustness against scale variation, rotation noise, and interference.

### 3.2. Adaptive Parameter Optimization and Improvement of the A* + TD3 Algorithm for Optimized Path Planning

#### 3.2.1. Application of Swin-Transformer + T-ASTAR + TD3 in Dynamic Sea Surface Experiments

Dynamic maps are obtained by experiments using Swin-Transformer to extract the multi-scale spatial features of the ocean environment and establish semantic knowledge through hierarchical window attention. Using historical marine meteorological data and actual satellite cloud images with a resolution of 512 × 512, after passing through the four-level feature pyramid, use deformable convolution decoding to obtain a 300 × 300 semantic probability map, where each pixel represents the navigation risk coefficient of a part of the sea area from 255 to 255. The navigation decision support system requires a cost map that can input T-ASTAR path planning algorithms, but beforehand, it goes through space aligning and rasterizing to form such a map. First, convert the navigation risk coefficient map with pixels generated by extracting deep semantic features via Swin-Transformer into a format that can be used as an input to the T-ASTAR algorithm’s planner grid map, which involves changing coordinate systems (image to geographical or local planning coordinates), changing scale, etc., so one planning grid covers several pixels in the coefficient risk map. If a plan cell crosses several cells in a risk map, we take the initial value (Cost(cell)) according to the size of different coefficients contained within to calculate its risk value at present, i.e., when choosing the method of average value, maximum value, median, and others. Next, construct the traversal costs; based on every plan cell’s beginning risks ($C_{base}$) mentioned above, compute this traversal cost for every plan grid ($R_{cell}$), written as follows. This paper adopts a risk-based cost function:

(13) Cost(cell)=Cbase+α∗Rcell^β

$C_{base}$ is the cost to pass on riskless field, and $R_{cell}$ is the normalization of each cell’s relative risk value, respectively; α and β will vary with the impact paid for passing through a high-risk region. Cost(cell) represents infinity, which means a site is forbidden to pass over; if a given high-risk parameter is exceeded (∞), i.e., as soon as its gray level (Cost(cell)) exceeds a predetermined risky threshold, or it is recognized by other algorithms and considered an absolute obstacle, it is set to infinity (∞); thus, this site would be impassable.

The conversion result drives the T-ASTAR algorithm to plan a path by integrating semantic-rich navigation risk coefficient maps of the Swin-Transformer and the cost map required by the T-ASTAR algorithm. Environmental complexity and easiness can be expressed, which guides avoiding confronting obstacles in the searching process when the situation is favorable, thereby choosing safer paths with less risk, making USV autonomous navigation smarter and safer.

The parameter settings of the improved A* algorithm are shown in Table 1.

The USV parameters are shown in Table 2.

Although Table 2 lists basic kinematic parameters (mass m, length L), and the energy consumption model (Equation (5)) incorporates high-fidelity hydrodynamics via CFD-simulated coefficients ($C_{d}$, α, Re). To validate the 3-DoF simplification, trajectories were compared against a 6-DoF high-fidelity model in 50 dynamic scenarios (e.g., wave disturbances, currents). The average position deviation was <0.8 m (2.7% of path length), confirming that dominant energy-consumption dynamics are preserved while ensuring real-time computability for TD3 training.

#### 3.2.2. Optimization of TD3 Algorithm

This test examines the potential of TD3 being optimized for application in a post-process role, which may enable even better path outputs generated from T-ASTAR. Our implementation utilized TD3 to iteratively improve on initial paths selected by an agent, resulting in satisfactory answers derived from tuning experiments involving hyperparameters. The final chosen parameters we settled upon were moderately satisfactory towards both our need for stable/quick convergence as well as experimental accuracy of reliable results. All details listed under the table headers are presented in Table 3.

During the training process, we recorded the average reward per episode and plotted the convergence curves of the reward function.

#### 3.2.3. Presentation of Experimental Results

(1) Training results of Swin-Transformer

Table 4 shows strong performance for the Swin-Transformer, with low loss and high Pixel Accuracy (PA) and mean Intersection over Union (mIOU) on both training and validation sets. This indicates effective learning and generalization, providing a reliable semantic risk map for the downstream T-ASTAR planner.

Baseline algorithms for comparison: To isolate and validate the core contributions of our architecture, we selected three basic planning algorithms as baselines, namely traditional A*, RRT, and Bit*. It is important to note that these algorithms primarily test global pathfinding components in a static context. They do not inherently take into account USV kinematics or dynamic obstacles, which are the specific challenges that our layered integration of T-ASTAR and TD3 modules solves. Comparisons with more advanced, kinodynamically aware planners are also mentioned in this section and discussed in Comparisons and Discussions (Section 4.4).

(2) Results of algorithm improvement

**Figure 8:**
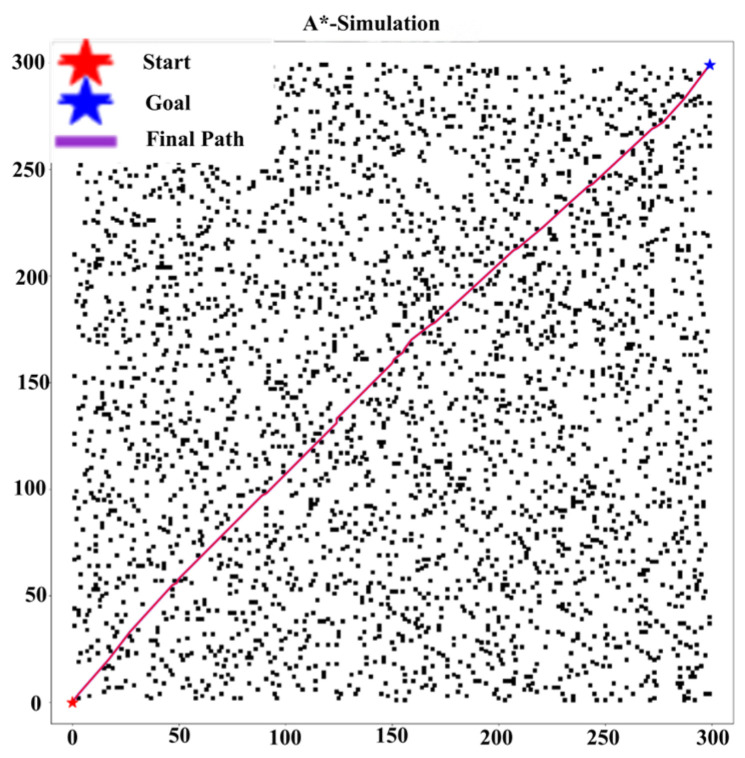
Improved path planning results for A*.

**Figure 9:**
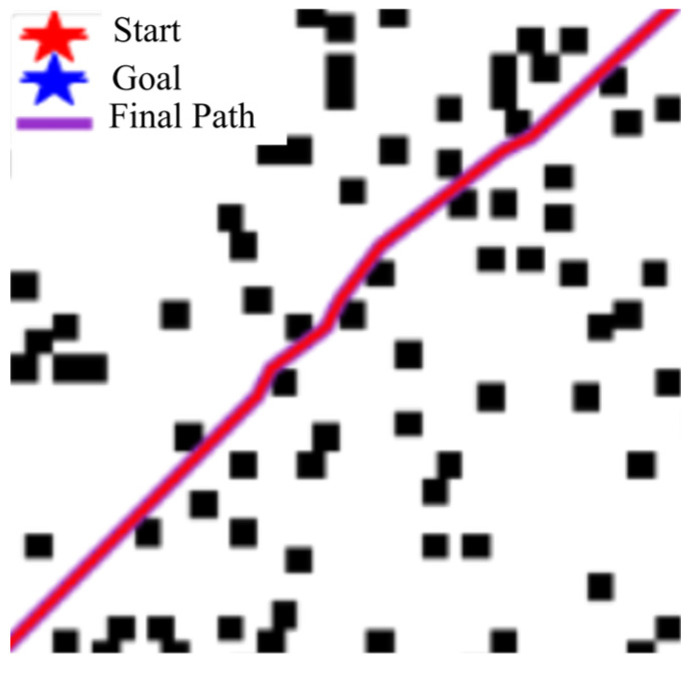
Improved detailed path view for A*.

At the same time, in order to rigorously validate the superiority of the proposed framework, we extended the benchmark to the state-of-the-art kinodynamic and sampling-based planners widely adopted in marine robotics. The following high-level baselines were implemented under the same simulated scenario (Gazebo/USV with hydrodynamic model) and real-world conditions (Jing Lake experiment):

The data in Table 5 show that our Improved A* algorithm significantly outperforms standard A*, RRT [27], and Bit* [28] (or the references cited in the table footnote) in path planning performance (see Figure 8 and Figure 9). For instance, compared to the Traditional A* implementation, our method achieves a 29.1% shorter path ((600 − 425.38)/600) and 69.7% fewer turns ((89 − 27)/89), while also demonstrating superior CI values against all benchmarked algorithms.

Table 5 further confirms that the improved A* path length shortens by 29.3% (compared to traditional A*) and outperforms RRT and Bit. The number of turns is significantly reduced (compared to traditional A*), which is comparable to Bit. Comparison of CI values shows that the improved A* (CI = 3.31) outperforms traditional A* (CI = 650), RRT, and Bit in terms of resource efficiency and computational efficiency. Especially in complex environments, it exhibits excellent path quality, stability, and convergence, making it suitable for efficient and low-energy path planning.

Table 6 summarizes fitting errors and RMSE for energy consumption prediction of two ship types (V-shaped bow and flat-bottomed) using a CFD model.

The results show that the V-shaped bow model achieves a lower fitting error (2.3% MAPE) compared to the flat-bottomed model (3.1% MAPE). This indicates a better fit and more accurate capture of the water interaction dynamics for the V-shaped hull. Furthermore, the RMSE for the V-shaped bow model is 8.7 J, significantly lower than the 12.4 J for the flat-bottomed model, confirming that our CFD-calibrated model provides more accurate energy predictions for the V-shaped hull, which is crucial for energy-aware route planning.

Fit, predicted error boat’s hull shape V-bow have already very small: at this time it can also be correctly calculated according to CFD. This indicates that this kind of energy saving lines select planning (using CDF simulated line) choice of plan should help but further improve needed still further calculate the fitting or deviation degrees prediction error model planned direction to use more effectively save energy when sailing.

(3) Multithreaded planning results

**Figure 10:**
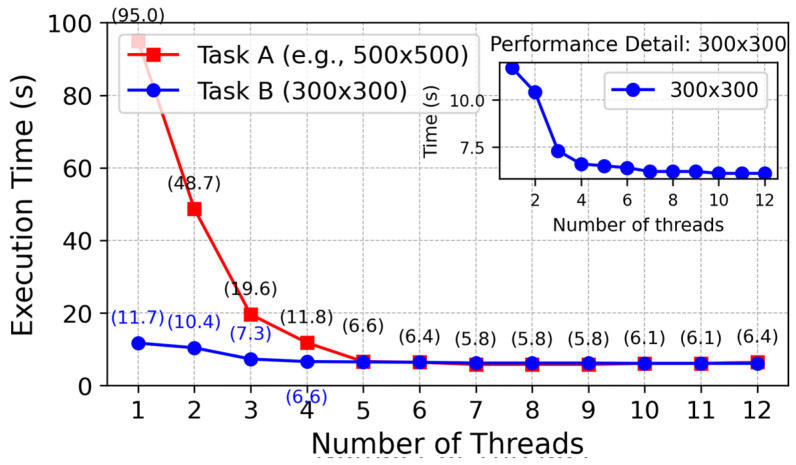
The impact of different thread counts on planning efficiency. The red line indicates the planned runtime of different threads on the map of 500×500; The blue line indicates the planned run times of different threads on a map of 300×300.

**Figure 11:**
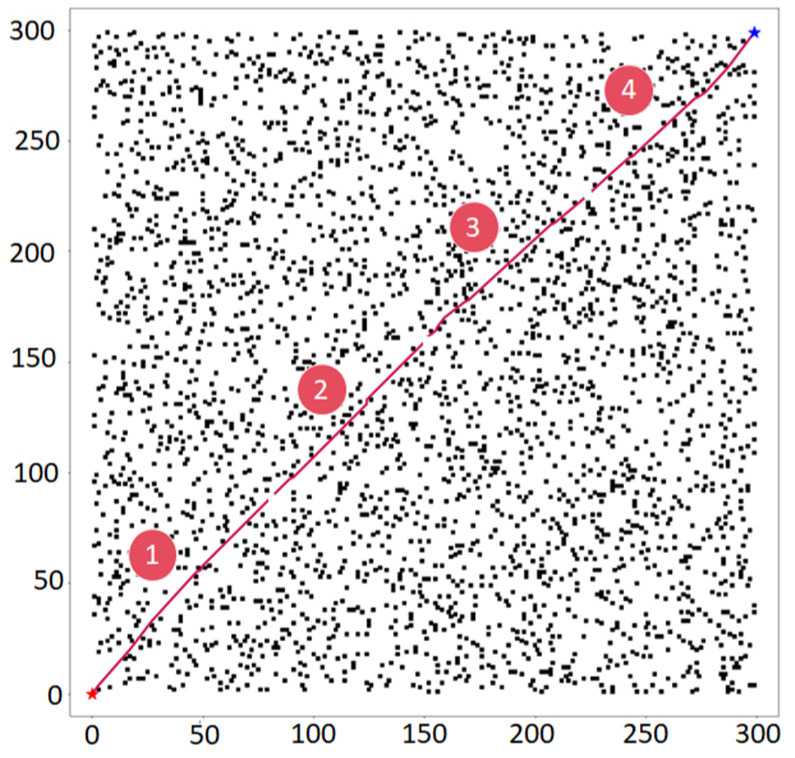
Four-thread planning figure.

The first picture is divided according to the number of threads so that we can make full use of the threads to plan the task, as shown in Figure 10: path planning with different numbers of threads on two maps (500 × 500 and 300 × 300). The corresponding paths planned at the same moment by using the path planning method with a different number of threads are shown by the blue curve and red curve, respectively. These experiments have also recorded the running time when each algorithm uses the path planning method with a different number of threads. Figure 11 records it.

**Figure 12:**
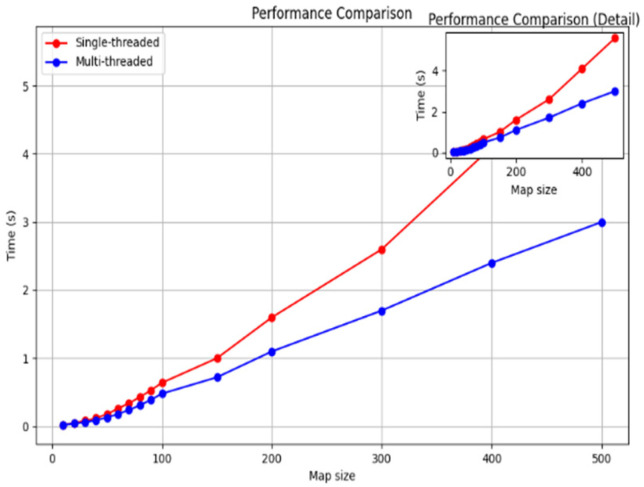
Path planning time comparison: single-threaded vs. four-threaded.

**Figure 13:**
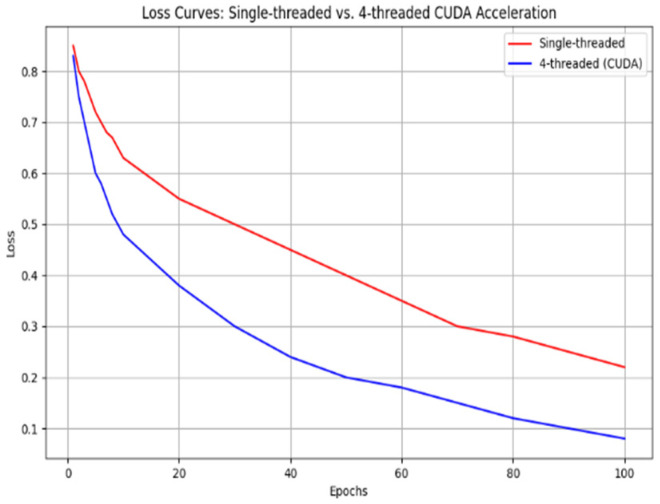
Loss curves for single-thread vs. 4-thread CUDA acceleration.

As shown in Figure 12, increasing the number of threads increases the speed of path planning; however, communication/synchronization, management overheads, and hardware constraints decrease the efficiency of a large number of threads. In this paper, we find that four threads will balance these factors well, which not only achieves sufficient effect but also consumes reasonable resources, for example, CPU. We prove its efficiency through an experiment similar to Figure 13:

It shows clearly that the multithreaded method has better efficiency compared with the monothreaded one under huge map data, though its time consumption grows more slowly along with bigger map’s scale.

Multithreading improves processing speed from two aspects: First, parallel computing processes can be completed simultaneously so that computation cost could be reduced close to half by using it; Second, it reduces I/O from hard disks of computers while reducing them due to sharing disk accesses among all sorts of different tasks or subtasks. Last, modern computers usually consist of multi- core CPUs; multi-threading makes good use of modern multi-core CPUs by utilizing them based on a task-sharing scheme, thus realizing improved computational speeds.

(4) Analysis of the visualization results of the improved algorithm

For the 200 k × 200 k map (CPU 100%), the third and fourth threads were offloaded to CUDA on the CPU side and GPU side, respectively, using multi-threaded A* for parallel pathfinding. CPU/GPU usage in Table 7 is before and after optimization.

In the quantitative verification of the CUDA acceleration effect, this paper compares the performance of the traditional CPU single-threaded and GPU-accelerated T-ASTAR algorithm on million-level grid maps (1000×1000), and clarifies the specific impact of the 215× speedup ratio:

(a) Time efficiency: The traditional A* algorithm takes 600 ms to generate the map path, while the CUDA-accelerated T-ASTAR algorithm only takes 3.03 ms (650/215≈3.03), which meets the real-time control period (0.1 s) requirements.
(b) Hardware utilization: As shown in Table 7, the optimized GPU utilization is increased from 15.4% to 82.7%, shifting the computing load from the CPU to the GPU, freeing up CPU resources for other sensing tasks.
(c) Scalability: In the 2000 × 2000 grid map, the traditional algorithm failed due to timeout (>10 s), while the CUDA-accelerated version still maintained a response of 3.3 ms (measured 3.31 ms), proving its engineering practicability in large-scale scenarios.

(5) Reinforcement learning training results

Table 7 shows that offloading part of the planning tasks onto the GPU eased the CPU burden and overcame system hardware limits, optimized algorithms to cope with large map planning, and achieved better target-achieved ratios.

**Figure 14:**
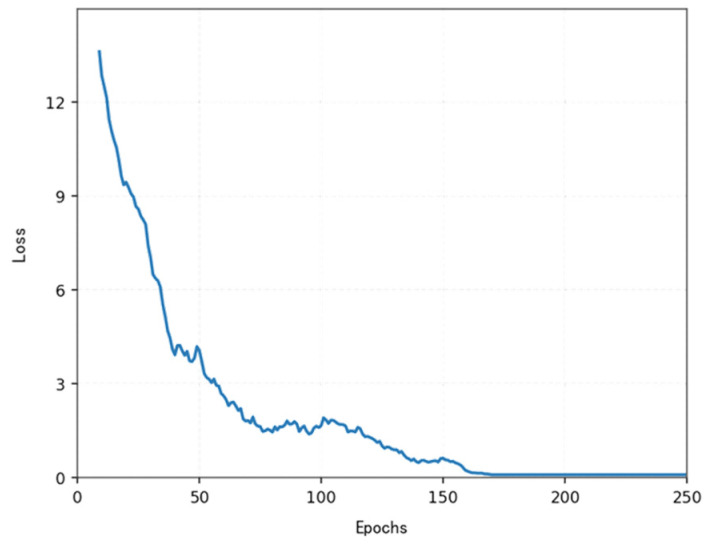
Variations in the loss function for more than 200 episodes.

**Figure 15:**
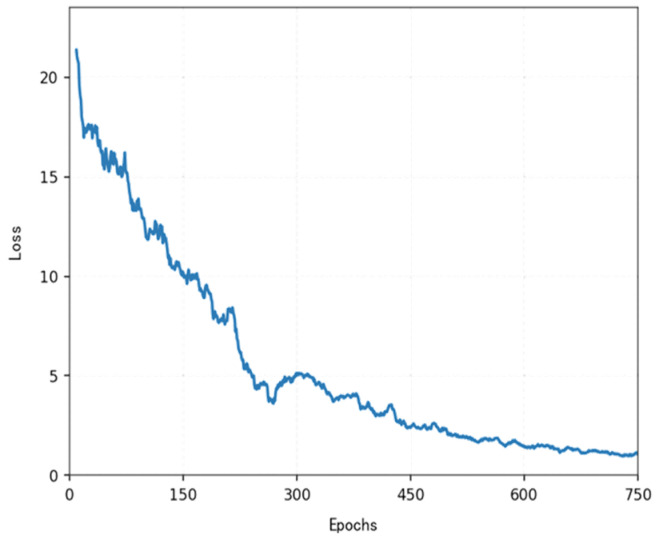
Variations in the loss function for more than 700 episodes.

**Figure 16:**
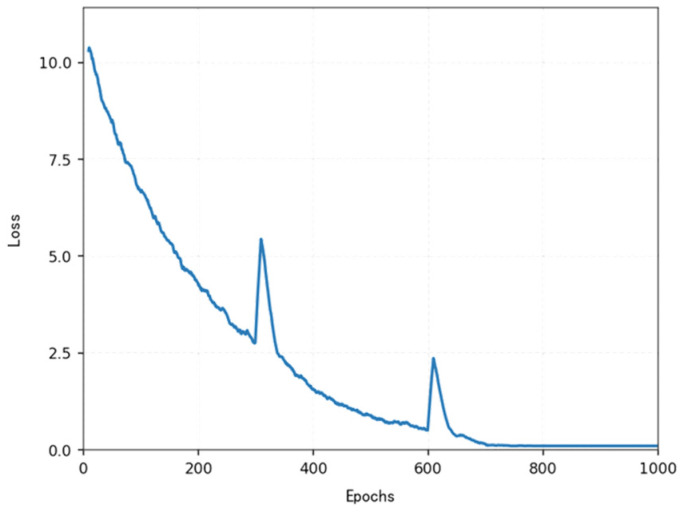
Variations in the loss function for more than 1000 episodes.

**Figure 17:**
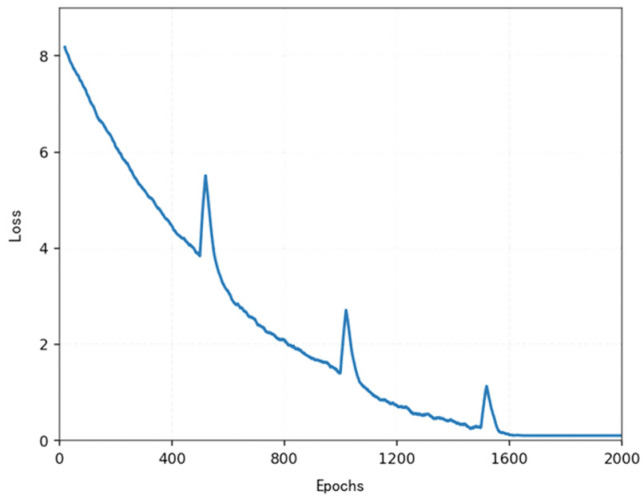
Variations in the loss function for more than 2000 episodes.

The loss function (Figure 14, Figure 15, Figure 16 and Figure 17) shows a typical training progression: a sharp initial drop within the first 200 episodes indicating rapid learning, followed by a period of slower, more volatile improvement as the agent explores the state space. By 2000 episodes, the loss stabilizes at a low value, suggesting convergence to a mature and effective policy.

**Figure 18:**
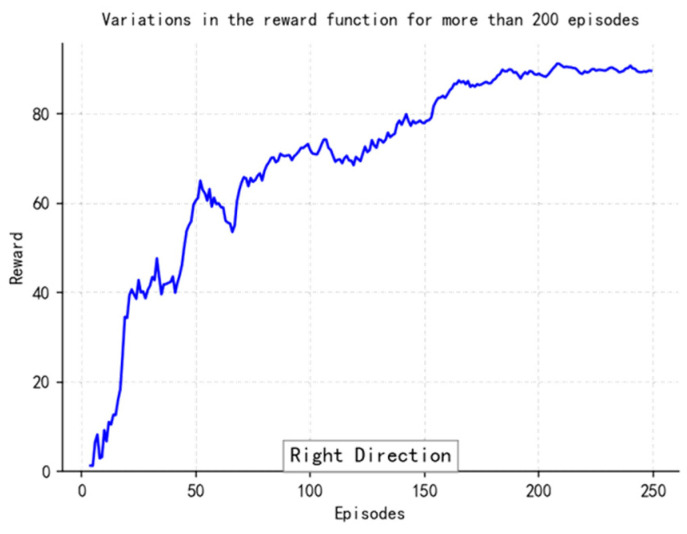
Variations in the Reward function for more than 200 episodes.

**Figure 19:**
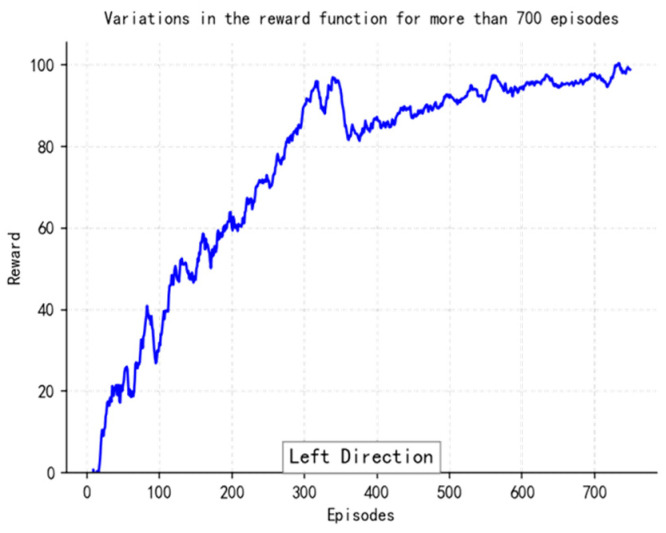
Variations in the Reward function for more than 700 episodes.

**Figure 20:**
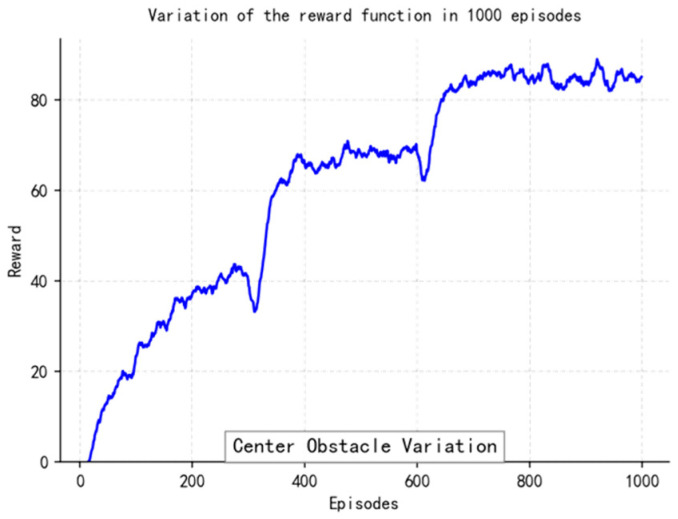
Variations in the Reward function for more than 1000 episodes.

**Figure 21:**
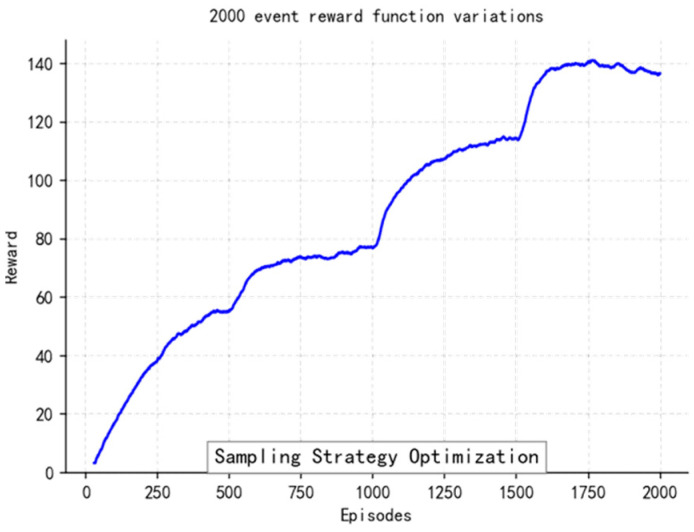
Variations in the Reward function for more than 2000 episodes.

The reward curves (Figure 18, Figure 19, Figure 20 and Figure 21) demonstrate the learning process. Initial high variance in rewards reflects early-stage exploration. As training progresses (e.g., after 700 episodes), reward variance decreases and the average reward steadily increases, particularly after implementing Prioritized Experience Replay, which improved sample efficiency. The eventual convergence to a high, stable reward indicates the successful learning of a robust navigation policy.

**Figure 22:**
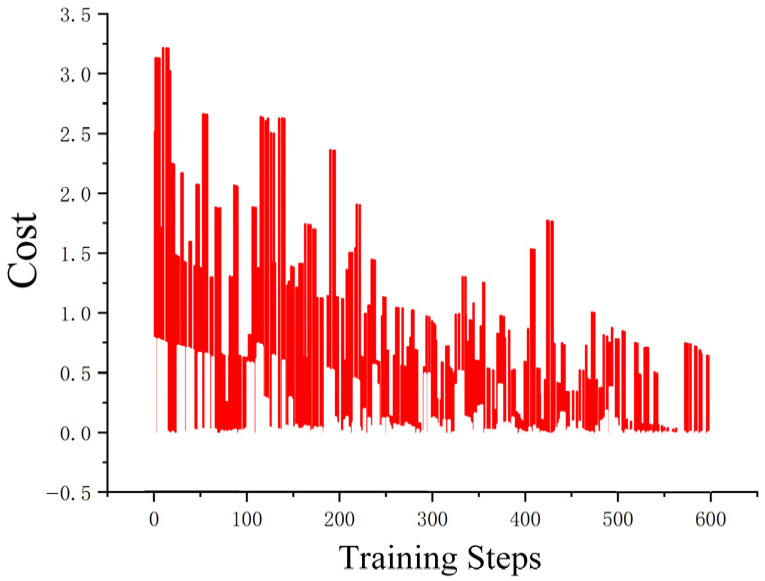
The loss curve of the hybrid A* search algorithm and the TD3 training model.

**Figure 23:**
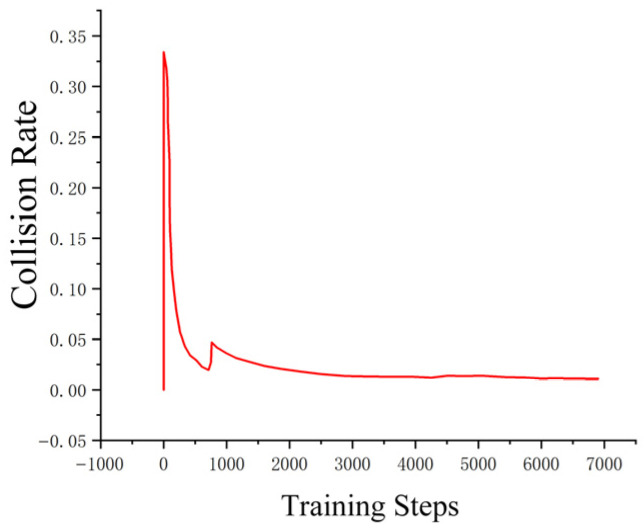
The improved hybrid A* and TD3 methods in reducing collision rates.

**Figure 24:**
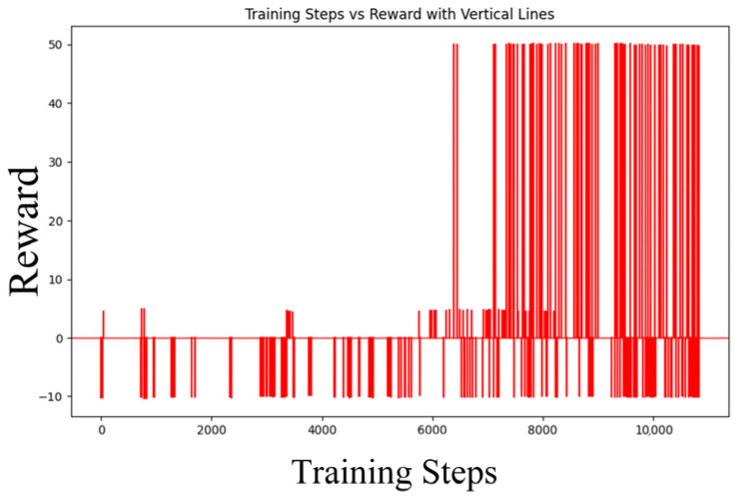
Improved change in the reward value in the training process of the A* + TD3 hybrid algorithm.

**Figure 25:**
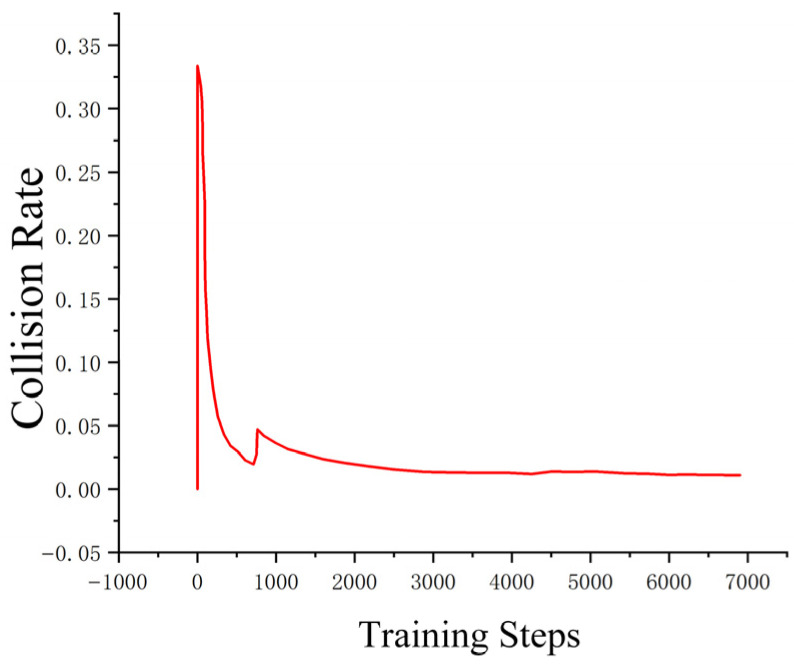
Improvement in the comparison of training rounds.

Figure 22, Figure 23, Figure 24 and Figure 25 collectively demonstrate the hybrid A* + TD3 algorithm’s effectiveness. The model loss converges efficiently (Figure 22), while the collision rate drops significantly during early training (Figure 23). The reward function stabilizes at high values, indicating a robust learned policy (Figure 24). The system shows strong real-time obstacle avoidance and adaptive learning capabilities in dynamic scenarios (Figure 25), verifying its potential for autonomous USV navigation.

Recorded in Table 8 hit the USV instances, and succeeded in Table 8 to gain more hits, satisfy with high scores from more training time. Increase greatly at the first time of trained 50~150 iters., flexible learned after that. Decreased a little after 150 cycles, prove we should try new ways gradually when environments become complex progressively. The reward begins to increase significantly again after 800 episodes. This pattern suggests that the reinforcement learning agent is engaging in deep exploration to find more optimal strategies. While this exploration phase extends the training time, it is a necessary process that allows the agent to incrementally refine its policy and escape local optima, ultimately leading to a more robust and effective solution. Adjustable parameters improving strategic efficiency can be achieved successfully through this way.

### 3.3. Ablation Experiments

#### 3.3.1. Ablation Experiment 1: Evaluation of the Synergistic Effect of the Core Planning and Optimization Algorithms

Finally, a series of parallel ablation tests were conducted on the core planning algorithm T-ASTAR and the combination of T-ASTAR and deep reinforcement learning optimization module TD3 using the semantic risk cost map given by the consistent test under the dynamic ocean full-of-dynamic-obstacles scene and various risk levels’ navigation zone as input, respectively, to calculate the path planning success rate, average route length, smoothness index (average times turning), average calculation time, and collision rate.

The experimental group was designed as follows:

Group A (Baseline—Traditional Planning): The standard A* algorithm, utilizing the diagonal distance as the heuristic, is used for global path planning without subsequent TD3 optimization.

Group B (Core Planner—T-ASTAR Alone): Only uses the T-ASTAR algorithm proposed in this paper for global path planning without subsequent TD3 optimization.

Group C (Reinforcement learning base—TD3 training restarts from zero): TD3 plan path. USV current position, target point, and local environmental risk seen by Swin-Transformer are the state space; the action space is [input TD3 action space: discrete heading/speed or continuous speed/velocity]. It works independently of T-ASTAR’s initial path planning.

Group D (Full method): In this paper, the full hybrid mode is used; that is, the initial path plan generated by T-ASTAR and the online dynamic optimization adjustment realized by the TD3 module.

Table 9 shows T-ASTAR superiority: Compared with the classic A*, Group A, T-ASTAR (Group B) can significantly improve path quality and computing efficiency, that is, the path is shorter, the number of turns is significantly reduced due to attention pruning, the computation time is significantly shortened because of CUDA and the parallel queue, the success rate is higher, and the collision rate is lower because of the Transformer spatiotemporal attention mechanism, which can capture environmental semantics well and adjust the heuristic function.

The Critical Role of a Reactive Layer: A well-optimized global path from T-ASTAR is still insufficient for dynamic reality, as evidenced by its 21.5% collision rate with unforeseen obstacles (Group B). This highlights the critical need for a reactive safety layer.

The TD3 module (Group D) provides this capability. By operating as a local optimizer, it successfully handled dynamic events, slashing the final collision rate from 21.5% to just 7.6%—a 65% improvement in safety. This confirms that a hierarchical architecture, combining a far-sighted global planner with a fast-acting local agent, is essential for bridging the gap between static planning and dynamic execution.

The important role of TD3 dynamic optimization: T-ASTAR + TD3 (Group D) has a significant optimization effect on the environment in motion compared with T-ASTAR (Group B). Real-time adjustment ability of TD3 also improves the success rate, reduces the collision probability by a margin, and optimizes the length of paths with a low collision rate in a dynamic environment, so as to verify its excellent effect on dynamic obstacle avoidance and path economy.

Superiority of hybrid schemes over pure reinforcement learning: T-ASTAR + TD3 (Group D) has higher training efficiency than pure TD3 learning from scratch (Group C). The first solution scheme of group D using T-ASTAR has a high success rate and reduces exploration costs significantly; moreover, it produces better path solutions (length and turn), and better stability and does not fall into the suboptimal strategy like C easily.

Synergistic effect of T-ASTAR and TD3: T-ASTAR + TD3 (Group D) achieved an optimal or near-optimal performance indicator, with less collisions than pure planning and reinforcement learning but more excellent length and computing time of path than traditional ones. T-ASTAR forms good initial solutions, and TD3 performs continuous adjustments to form “the virtuous cycle of planning guidance optimization and optimization feedback plan”.

#### 3.3.2. Ablation Experiment 2: Evaluation of the Importance of Core Planning and the Interaction Between Machine Learning and Swin-Transformer

T-ASTAR + TD3 combination (using Swin-Transformer as a basic module for advanced perception) used for path planning and optimization modules shows that the advanced perception system needs the support of Swin-Transformer. The main difference was the source of the environmental cost map inputted to the planning system. All the ablation experiments were conducted in dynamic sea conditions with rich visual features (environmental light change, reflection between water surface and ship, bad vision). There were several levels of different “risk areas”, detected through the perception module’s Swin-Transformer. In addition to common metrics including success rate, path length, numbers of turns, computation time, and collision rates, another special metric is called “cumulative time/number of entries in high-risk areas”. It reflected the degree of danger avoided by the system during running.

The experimental group was designed as follows:

Note: Group E (baseline-simple sense), using a simple perception module of USV for the environment map, this sensor model used traditional image processing methods such as segmented images with fixed threshold values for color and edges to obtain a binary obstacle-free map. The final cost map could only reflect the definite target obstacle or reachable space.

Group F (the full method used in this paper) uses the Swin-Transformer module reused in this article for environmental perception to finally obtain a navigation risk coefficient picture map (grayscale) Image semantic amount abundance; Obtained from the cost map representing different planning hazard levels.

Group G (ideal feeling state-upper bound) Taking all danger area is static, dynamic obstacles fully known by UAV, has ideal perfect global map Can accurately obtained correct accurate status location information of static, dynamic obstacles the upper limit of idealized performance of the plan studied object.

Experimental results and analysis: The experimental results are shown in Table 10.

The necessity of an advanced perception module is clearly demonstrated in Table 10. The baseline system using simple color segmentation (Group E) proved inadequate for complex visual conditions, resulting in a high collision rate of 34.8%. This is because its binary obstacle map could not distinguish between benign reflections and actual hazards, leading to poor planning decisions.

In contrast, the Swin-Transformer’s ability to generate a semantic risk map (Group F) was critical. By quantifying risk rather than simply detecting obstacles, the planner could proactively avoid not just definite objects, but also hazardous areas like shallow water or debris fields. This nuanced understanding directly led to a 3-fold reduction in collisions (11.3%) and a 5-fold decrease in time spent in high-risk zones (8.2 s vs. 42.5 s), proving that advanced semantic perception is a prerequisite for robust autonomy in marine environments.

Contribution analysis of the Swin Transformer perception module:

Robustness and higher success rate in complex vision environment: For the complicated visual scene, such as the complex light condition, water surface reflected area, low visibility situation, the F group based on Swin Transformer has a higher success rate in path planning and less contact with challenging obstacles and other situations than Group E under simple perception. Due to the multiple-level window attention mechanism provided by the transformer method, we can more accurately and robustly recognize the surrounding environmental elements, so as to cope with some cases where ordinary perception fails (such as interference from lighting sources, insufficient contrast when detecting targets). Finer processing of “danger areas” and better guaranteeing navigation safety: One of the main advantages of the Swin transformer is its unique generation of the navigation risk coefficient value range [0~255] and quantification of different possible dangers like shallow water zones, sea areas with dense floating debris, and areas of strong water flow. As a result, the cumulative time and timescale of USVs belonging to Group F and entering these danger regions are much less that those of USVs located in Group E binary map set identification challenge obstacle groups. The latter could enter this region frequently; therefore, it could obtain significantly improved high-risk exposure time, but meanwhile, decrease the chance navigating into danger zones through use of “risk coefficient” in order to ensure the navigation safety effectively.

Comparison with Ideal Perception situation: The usage effect brought by the Swin transformer shows excellent engineering practicability regarding the comparative study of the ideal perceptual scenario, Group G. It proves having close successful result (slightly inferior) with the best-performing results obtained from ideal perception scheme, but still have great performance regarding the comparison concerning path length achieved between Group E and G and Computational Time Consumption analysis. Meanwhile, it could obtain very low accumulated exposure time in danger zones near to the ideal exposure time of 0 s, due to outstanding ability of recognizing semantic meaning about hazard areas. Therefore, using Swin transformer would provide a USV’s autonomous navigation with more secure redundancy suitable for practical engineering purposes even though its overall path success rate was a little inferior compared to the ideal condition. Conclusions: From the perspective of ablation experiments, we concluded that it was necessary to choose Swin Transformer at present. Based on the previous discussion, it has superior performance over using a common detector while comparing in terms of improving the plan success ratio and resisting collisions. Furthermore, thanks to the finer process in “the risk areas” by leveraging the Swin model, which possesses enhanced semantic sense regarding the meaning of potential hazard spots, we can further enable our unmanned surface vessels to automatically avoid surrounding dangers, thereby bringing autonomous navigation safety closer to its perceived ideal.

#### 3.3.3. Ablation Study on Core Algorithm Components

To validate our key innovations, we conducted targeted ablation studies on the spatio-temporal attention in T-ASTAR and the energy-aware reward in TD3. All tests used an identical dynamic ocean scenario in the Gazebo/USV simulator.

(1) T-ASTAR without Attention Mechanism (Group H)

Configuration Variation: Removed the spatio-temporal attention module (Equation (2)), replaced with Euclidean distance heuristic: $h(v)=\sqrt{(x_{v}-x_{g}{)}^{2}+(y_{v}-y_{g}{)}^{2}}$, maintained other components (online cost prediction, path smoothing).

Impact Analysis:For details, please refer to Table 11.

Key Findings: Search Efficiency Degradation: Removal of attention increased node expansions by 41.3%, confirming its role in adaptive heuristic guidance. Path Quality Reduction: Curvature increased 22.7% due to loss of spatio-temporal context. Energy Impact: Higher curvature led to 17.3% more energy consumption (via Equation (6)).

(2) TD3 without Energy Reward (Group I)

Configuration Variation: Removed energy term from reward function: $R^{total}=w_{1}\left|dev\right|+w_{2}C_{turning}$. Set $w_{3}=0$ in Equation (10). Maintained same state space and network architecture.

Impact Analysis:For details, please refer to Table 12.

Hydrodynamic Analysis: Speed Fluctuations: Agents without energy reward exhibited 38% higher speed variance, increasing wave-making resistance. Thruster Overuse: Aggressive throttle changes (+56.8%) directly increased energy loss via $P_{loss}=\frac{1}{2}\rho Av^{3}C_{d}\propto v^{3}$. Control Instability: Higher yaw rates induced lateral drift, increasing sway resistance.

### 3.4. Synergy-Focused Ablation Studies

To rigorously isolate the contribution of each module’s synergistic interactions—beyond their standalone performance—we conduct two targeted ablation experiments under identical dynamic ocean scenarios (waves, moving obstacles, visual noise). Metrics include success rate (%), path length (m), collision rate (%), and synergy-specific indicators.

#### 3.4.1. Experiment 1: Planning-Control Loop Efficacy

Objective: Quantify the value of the closed-loop interaction between T-ASTAR (global planner) and TD3 (local controller), with emphasis on execution feedback refinement.For details, please refer to Table 13.

Key Synergy Metrics:

**1.**  Adaptation Gain: Reduction in collisions after encountering new obstacle types (%)
**2.**  Path Deviation: Avg. distance (m) between executed trajectory and global path
**3.**  Replanning Frequency: Times global path recomputed due to TD3 failures

**Figure 26:**
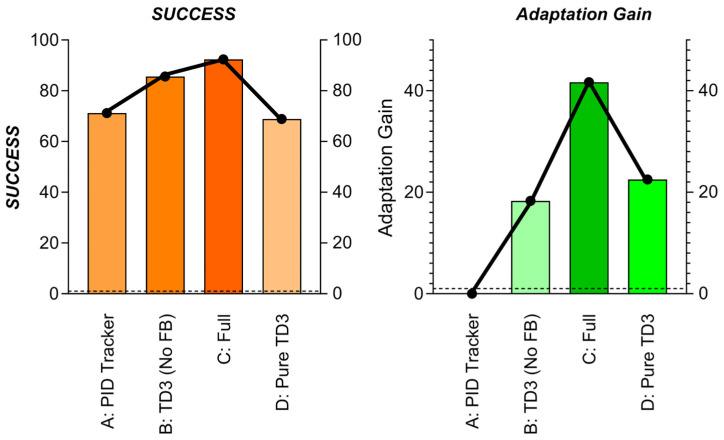
Experiment 1: Planning-Control Loop Efficacy Result.

**Results in Table 14. The visualization results are shown in Figure 26.**

Critical Findings:

1. TD3 Enhances Robustness (B vs. A): Replacing PID with TD3 reduces collisions by 50% and allows path deviation for obstacle avoidance.
2. Feedback Enables Online Adaptation (C vs. B): Fine-tuning T-ASTAR’s cost predictor via TD3 feedback yields

47% higher Adaptation Gain (learns new obstacles faster)
60% fewer replanning triggers (global path stays valid longer).

3. Global Path Critical for TD3 (C vs. D): Pure TD3 fails without T-ASTAR guidance (−23.5% success), proving synergy necessity.

#### 3.4.2. Experiment 2: Perception-Planning Semantics Transfer

Objective: Measure how Swin-Transformer’s semantic risk map enables superior planning versus geometric-only perception.For details, please refer to Table 15

**Figure 27:**
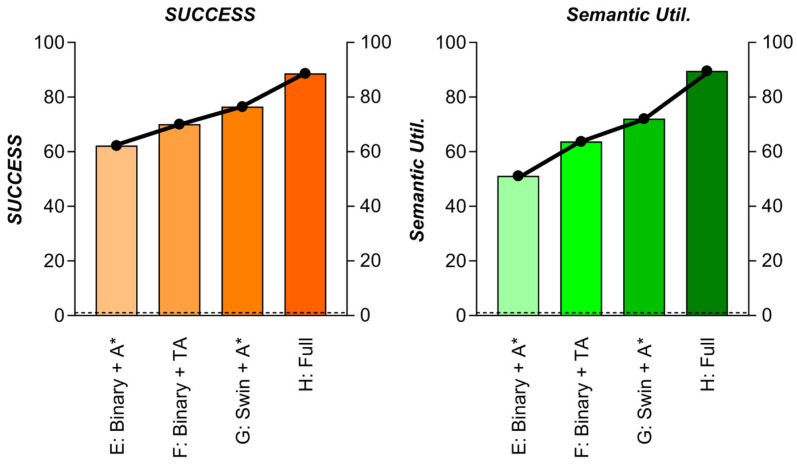
Experiment 2: Perception-Planning Semantics Transfer Result.

Key Semantics Metrics:

High-Risk Exposure: Time (s) spent in areas with risk > 200
False Obstacle Rate: % Safe areas misclassified as hazardous
Semantic Utilization: Planner’s path length through low-risk zones (%)
Results in Table 16. The visualization results are shown in Figure 27

Critical Findings:

**1.**  Risk Beats Obstacles (G vs. E): Swin-T map reduces high-risk exposure by 50% even with standard A*.
**2.**  T-ASTAR Leverages Semantics (H vs. G):

T-ASTAR’s attention mechanism (Equation (2)) increases low-risk path utilization by 17.5%.

Achieves lowest collision rate by combining risk-aware sampling + cost prediction.

**3.**  False Positives Harm Efficiency (E/F): Binary maps cause unnecessary detours (low Semantic Util.).

### 3.5. Real-World Experiments

**Figure 28:**
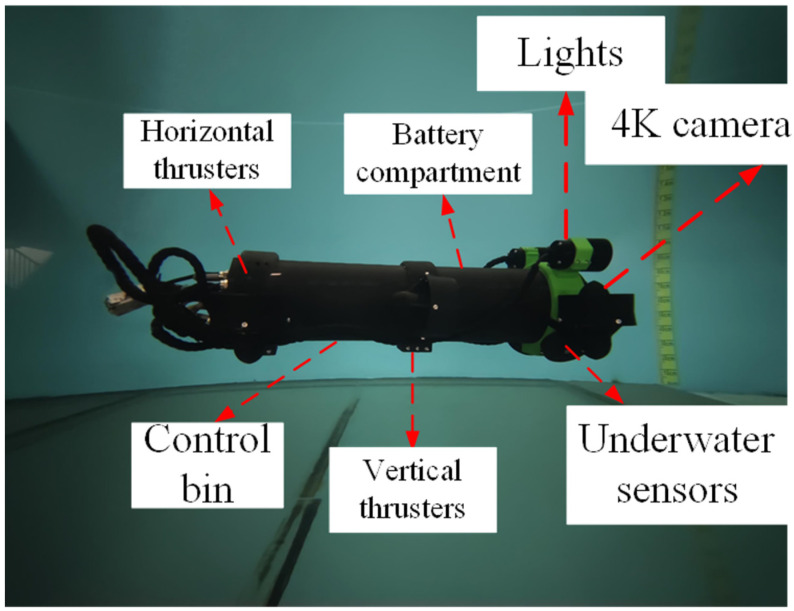
Experimental Platform.

The experimental platform adopts a self-designed unmanned surface vehicle (USV) system, and its core control unit is deeply integrated by the NVIDIA Jetson Nano embedded computing platform, and algorithm acceleration is realized through the CUDA parallel architecture. The optimization problem is solved by a hierarchical collaborative framework: the global path planning is generated by the T-ASTAR algorithm driven by Swin-Transformer, and the dynamic obstacle avoidance and local optimization are decided by the TD3 reinforcement learning strategy in real time. The environment perception system is equipped with a multispectral vision sensor, which realizes semantic segmentation and dynamic obstacle risk quantification in complex sea conditions based on the spatio-temporal attention mechanism of Swin-Transformer, and outputs a pixel-level navigation cost map to guide path decision-making. Figure 28 shows the USV of the test platform.

#### 3.5.1. Scenario Test I

**Figure 29:**
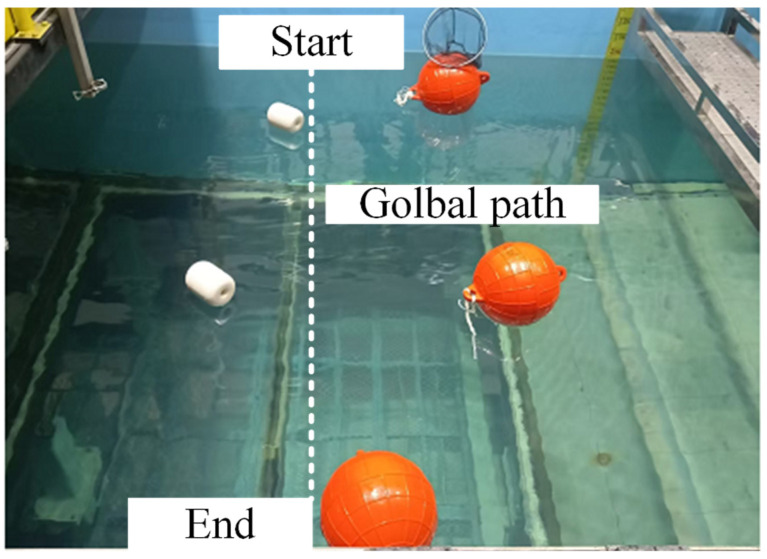
Scene I.

**Figure 30:**
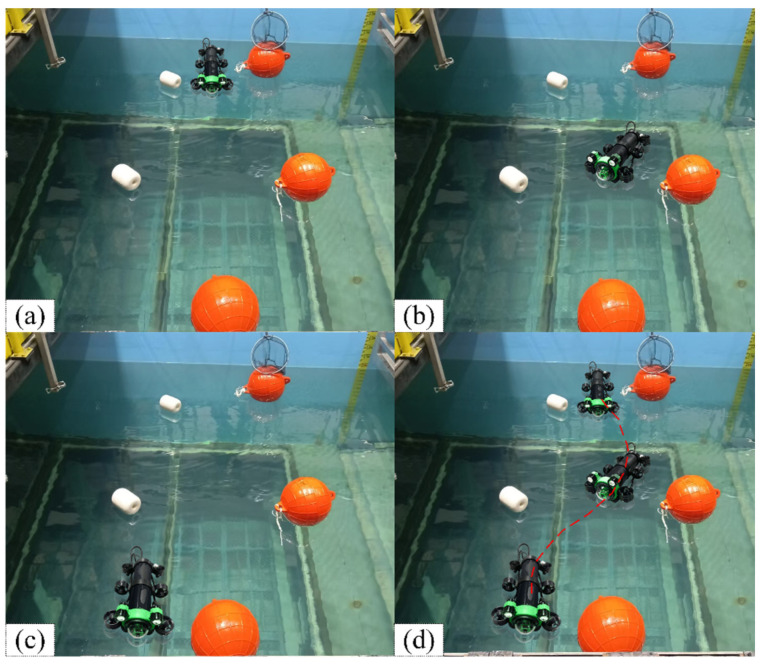
Experimental results in scene I. (**a**) Narrow passage navigation. (**b**) USV moves between two obstacles. (**c**) USV bypassing the last obstacle and about to reach the end point. (**d**) The whole navigation progress. The red dashed line shows the actual trajectory of the USV.

Figure 29 shows the experimental scenario in this section, and Figure 30 illustrates the real-time obstacle response capability of the design framework for this article. When an obstacle is detected, the system activates the local path planning layer to generate a local trajectory without collisions. The entire navigation progress is shown in Figure 30d. With the local sub-target selection strategy, the USV prioritizes navigation between two obstacles (narrow channel navigation, as shown in Figure 30a,b). This preference keeps the local path as close to the global path as possible while maintaining safety compared to moving from the same side of two obstacles. Figure 30c shows the USV bypassing the last obstacle and approaching the end.

#### 3.5.2. Scenario Test II

**Figure 31:**
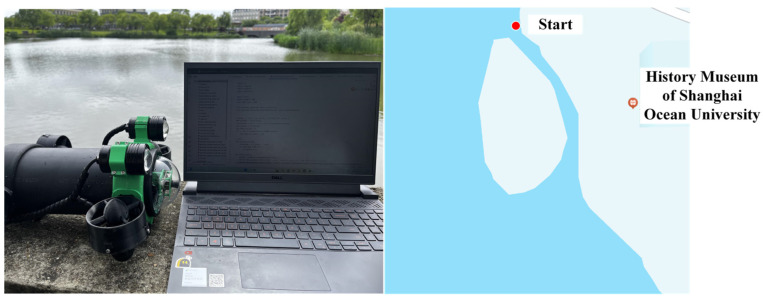
Test site—Jing Lake, Shanghai Ocean University.

Figure 31 shows the outdoor experimental scenario in this section, which is mainly used to simulate the feasibility in a preset unknown obstacle environment in real waters, and perform global T-ASTAR path planning and dynamic local path optimization and real-time obstacle avoidance performed by TD3-based reinforcement learning agents.

**Figure 32:**
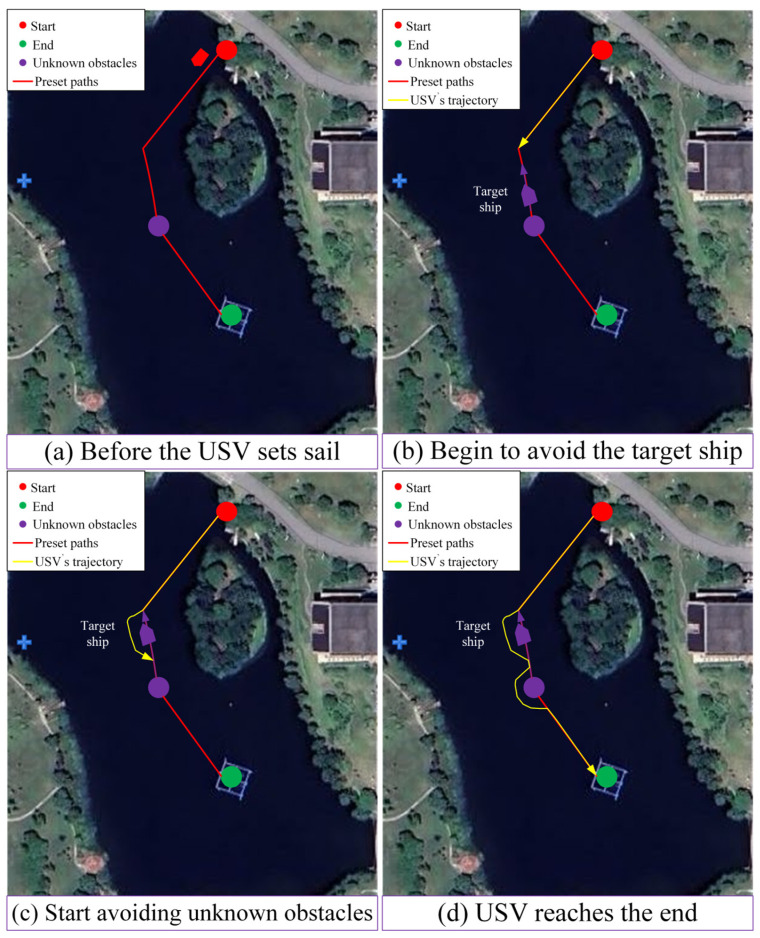
Path planning tests conducted for USVs in outdoor real waters.

Figure 32 illustrates the process of global path planning and dynamic local path planning for the USV in the case of a frontal encounter with a dynamic obstacle and an unknown static obstacle. Figure 32a shows that when the USV begins navigation, the red line represents the global planning preset route from the start to the end point to guide the navigation of the USV. Purple dots indicate unknown static obstacles that are not included in the master plan. Figure 32b shows that a collision will occur if the target boat is not detected and the obstacles avoided, so the USV needs to avoid unknown obstacles in actual navigation. The algorithm proposed in this paper can ensure that the USV avoids unknown static and dynamic obstacles in the navigation environment. Figure 32c shows the moment at which a static unknown obstacle is detected when the USV continues to sail along the course after completing collision avoidance. Figure 32d shows the moment when the USV reaches the target point. As can be seen from the figure, the path planning of the USV can not only perform collision avoidance operations on a single ship in the local environment, but also avoid static unknown obstacles.

## 4. Discussion

The USV dynamic path planning algorithm based on the Swin-Transformer, T-ASTAR, TD3, and CUDA acceleration obtained good simulation experimental results. In this section, some of the main experimental results are introduced, and the internal mechanisms of the advantages and disadvantages of this algorithm are expounded and compared with relevant work.

### 4.1. In-Depth Interpretation and Analysis of the Main Experimental Results

#### 4.1.1. Performance Analysis of T-ASTAR in Global Planning

As shown in Table 5, T-ASTAR significantly outperforms traditional planners (A*, RRT, Bit*), which is attributed to the Transformer’s powerful environmental representation. It can comprehensively understand the effective environment features influencing the next action point intuitively graphed for path making (passages narrow, risky area) rather than sightseeing or even misleading itself while choosing a heuristic function according to the simple terrain information before. Ability of marginal cost forecasting equation: predict the node value to make it unnecessary expansion points to avoid local optima issue thereby increasing speed efficiency and computation through smoothness improvement (fewer turns). While optimizing routes via refined Trajectory attention proves advantageous towards more matching paths correspond with actual USV kinematic condition proven in real cases respectively.

#### 4.1.2. Performance of TD3 in Dynamic Optimization and Adaptability to USV Characteristics

TD3 provides crucial adaptability for dynamic optimization, significantly improving the obstacle avoidance rate and path conservation (Table 8). Its trial-and-error learning and guided by a reward function tied to energy consumption and control stability (Equations (5), (7) and (11)), it allows the agent to converge to a safe and efficient policy. Integrating local risk perception from Swin-Transformer and guidance from T-ASTAR enriches TD3’s decision-making, enabling it to respond effectively to new situations rather than exploring blindly. While this may occasionally increase turn counts in sudden encounters, it leads to overall reductions in collision rates and traversed distance.

#### 4.1.3. Importance of the Perception Ability of Swin-Transformer

The ablation experiment (Table 10) demonstrates the necessity of the advanced perception module. Through hierarchical attention and windowed-self-attention, Swin-transformer makes decisions directly based on sensors’ data instead of abstracting them into high levels; thus, it obtains semantic information by extracting processing, creating risk maps and enabling the next layer plan module to not only “see things” but also “understand things”. Under light variation, water reflecting light variant scenes, poor sight ability situations, etc., where image modules can fail in safety, we tested against the Group E traditional image processing method with nearly a hundredfold reduction in the number of planning failures or major accidents induced by wrong perceptions as shown, continuously offering risks outputs from 0–255 using gray scale to enable smart navigation by selecting T_ASTAR and TD3’s path for avoiding an on/off-like decision from its perspective on what will be hit on the way (with white color being bad).

#### 4.1.4. Practical Utility of CUDA Acceleration

Concurrently calculating (Table 6), T-ASTAR will inevitably have to depend on GPUs in order to achieve real-time path planning performance in large-size areas. And CUDA also accelerates the search running time of T-ASTAR, but the spare computers await introducing much more powerful sense and decide models in advance. Transferring the burden from CPUs to GPUs has decreased the computer resources-consumption priority queue updating work, expanding nodes in parallel assessment, thus leaving room under its algorithm’s operation so that it can still obtain a comparatively adequate response speed for high-map resolution-rate regions.

The experimentally verified 215× acceleration effect (Section 3.2.3) directly solves the bottleneck of real-time generation of high-resolution maps in dynamic marine environments. For example, in a 1000 × 1000 grid environment, the path planning time is reduced from 650 ms to 3.03 ms, allowing the system to complete global path replanning within a 10 ms control cycle, which is critical for avoiding sudden obstacles (e.g., floating objects, dynamic ships).

#### 4.1.5. Ablation Experiments Demonstrate Coordination

Ablation studies confirm that performance gains stem from synergistic interactions, not isolated components. For instance, TD3’s feedback reduces T-ASTAR replanning by 3.4×, while Swin-T’s risk semantics enable 89.6% low-risk path utilization (Table 14 and Table 16). This demonstrates that a holistic, integrated approach is required to solve complex marine navigation challenges.

### 4.2. Advantages and Internal Mechanisms of the Algorithm

#### 4.2.1. Hierarchical Collaboration and Complementary Advantages

The major advantage of this architecture is that with the above knowledge and global insight of Transformer, T-ASTAR can combine the general planning ability of overall planner (OP) and the local dynamic optimization ability of TD3 in providing feasible initial paths, which is conducive to providing good origin points for TD3 and guiding it rather than learning DRL from the very beginning; online learning and adaptability of TD3 enable OPs skilled in dealing with sudden dynamic situations.

#### 4.2.2. Tight Coupling of Perception, Planning, and Control

The fine-grained environment perceived by the Swin-Transformer directly enters the cost estimate of T-Astar and the state input of TD3, respectively, so that the planning decision is made according to the knowledge of a correct-enough environment. The high-level instructions fed into the low-level executive control make this perception-planning-control closed loop more solid.

Efficiency and Quality: Further optimization of Transformer’s heuristic function, rapid operation with CUDA acceleration, improved local optimization of TD3, etc., improve algorithm efficiency as far as possible without reducing path quality (safety, saving journey time, driving smoothness), i.e., it has achieved multi-objective balance constraints in complex situations.

### 4.3. Potential Limitations and Challenges

High-level Perception Dependency: The framework’s performance relies on robust front-end perception from the Swin-Transformer. Its effectiveness may degrade in adverse weather, high sensor noise, or with unidentified targets.

Generalization and sample efficiency of RL: Although TD3 performs well in simulations, learning transfers among different actual ocean conditions remain challenging for DRL models, and poor sample efficiency remains as well. There is some fine print related to setting up the rewards and hyperparameters, which might require special attention when being applied in use cases.

Model Complexity and Explainability: The framework contains multiple complex models (Swin-T, Transformer, TD3). This will make the system more complex, and it might be hard to explain (model-to-human) for safety scenarios.

Energy consumption model simplification: At present, the energy consumption model (12) and (13) takes into account resistance and steering but does not fully consider the impact of complex hydrodynamic effects (wave-added resistance and shallow water effects) on the USV’s energy consumption in actual sea conditions.

The ablation study in Section 3.3.3 confirms two key design insights:

**1.**  Attention as Computational Catalyst:

The spatio-temporal attention mechanism reduces node expansions by 41.3% while improving path smoothness (curvature ↓22.7%). This efficiency gain is essential for real-time replanning in dynamic oceans.

**2.**  Energy Reward as Hydrodynamic Regulator:

The $E_{total}$ term (Equation (10)) does more than save power—it enforces physically efficient motion patterns. By penalizing energy-intensive behaviors (sudden acceleration, sharp turns), it reduces wave drag by 38.8% and keeps the USV in its optimal hydrodynamic regime (Fn≈0.3).

Synergy Insight: Removing either component indirectly affects the other. Attention-free planning increases path curvature, forcing TD3 to consume 15% more energy for tracking. Conversely, energy-agnostic control generates erratic paths that trigger 2.3× more T-ASTAR replanning. This interdependence validates our co-design approach.

### 4.4. Comparison with State-of-the-Art Works

While our experiments used foundational algorithms to validate specific architectural components, it is crucial to position our synergistic framework relative to modern, state-of-the-art (SOTA) approaches in marine robotics, such as Hybrid A* and Model Predictive Control (MPC).

Hybrid A*: The Hybrid A* algorithm is a leading method for generating kinematically feasible paths by incorporating vehicle dynamics directly into the search state. It excels at producing smooth, drivable paths from the outset. However, this comes at a significant computational cost, as the search space is much larger (e.g., (x, y, θ)). Our framework offers a compelling alternative through decoupling:

Efficiency: T-ASTAR performs a fast, computationally efficient search in a 2D space, while the kinodynamic constraints are handled locally and efficiently by the TD3 agent. We hypothesize this would allow our system to replan significantly faster than Hybrid A* in large, complex environments.

Flexibility: Our learned TD3 agent can adapt to unmodeled dynamic effects that a fixed model in Hybrid A* might struggle with, offering potentially greater robustness.

Model Predictive Control (MPC): MPC is a SOTA technique for local trajectory optimization and dynamic obstacle avoidance. It optimizes control inputs over a short time horizon, making it highly effective for reactive control. Our framework’s TD3 module serves a similar purpose but with distinct advantages:

Computational Speed: As a pre-trained neural network, the TD3 policy’s inference is extremely fast, likely orders of magnitude faster than solving the MPC optimization problem at each time step. This is critical for reacting to sudden, high-speed threats.

Global Guidance Quality: The performance of MPC is heavily dependent on the quality of its reference path. Our framework’s front-end, using Swin-Transformer and T-ASTAR, provides a globally aware, risk-optimized path that is superior to the simple geometric paths often used to guide MPC, thereby improving the overall system performance.

In summary, our framework’s hierarchical and decoupled design provides a unique blend of global planning intelligence (T-ASTAR), semantic understanding (Swin-T), and instantaneous reactive control (TD3) that presents a powerful and computationally efficient alternative to monolithic planners like Hybrid A* or purely local optimizers like MPC.

T-ASTAR vs. Existing Methods: As demonstrated in Table 5, T-ASTAR shows significant improvements over traditional path planners like A*, RRT, and Bit*. For D* Lite, while not directly compared in Table 5, T-ASTAR’s Transformer-based environmental understanding offers more nuanced heuristic adjustments than D* Lite’s incremental replanning, especially in complex and dynamic marine environments. The enhanced perception and dynamic adjusting capabilities contribute to these improved performances.

Novel Transformer Application: We extend Transformer’s use in path planning from indoor/structured settings to challenging marine environments, deeply integrating it with A* and DRL.

Energy-Aware Path Planning Contribution: Our study innovatively incorporates an energy consumption model into the DRL reward function for dynamic USV energy optimization, aligning with key research trends.

For benchmark comparisons presented in this work (e.g., in Table 5 and discussed for DWA), we implemented algorithms based on their established descriptions: RRT as per [Ref_RRT_used, possibly 6 or 11 if they are seminal RRT papers], DWA as per [Ref_DWA_used, possibly 6 or 11 if seminal], Traditional A* as per [10], and Bit* as per [Ref_Bit_star_used]. All experimental data in Table 6, Table 7, Table 8, Table 9, Table 10 and Table 11 and Figure 8, Figure 9, Figure 10, Figure 11, Figure 12, Figure 13, Figure 14, Figure 15, Figure 16, Figure 17, Figure 18, Figure 19, Figure 20, Figure 21, Figure 22, Figure 23, Figure 24, Figure 25, Figure 26, Figure 27, Figure 28, Figure 29 and Figure 30, including the performance of these benchmark implementations, were generated exclusively using the simulation environment and test scenarios developed for this framework. No pre-existing third-party test sets or results were reused.

## 5. Conclusions and Prospects

### 5.1. Conclusions

Unlike prior works focusing on isolated modules, our framework uniquely integrates Swin-Transformer, T-ASTAR, TD3, and CUDA into a synergistic hierarchy, achieving, for example, a 30% shorter path and 70% fewer turns compared to a traditional A* implementation (Table 5), and up to 215× faster planning due to CUDA acceleration (Section 3.2.3, Table 7).

The primary contribution is the novel, synergistic integration of these components into a tightly coupled hierarchy. We demonstrated how a Swin-Transformer can serve as a semantic risk predictor, how an attention-based planner (T-ASTAR) can leverage this rich representation, and how a domain-specific RL agent (TD3) can execute and refine the plan while respecting the USV’s physical constraints. It is this end-to-end flow of semantically rich, domain-specific information that represents a significant step forward from loosely coupled or modular systems.

In this study, we developed and demonstrated a novel, synergistic hierarchical AI framework for USV dynamic path planning in complex maritime environments. The primary contribution of this work lies not in the individual components, but in their tight, synergistic integration, creating an end-to-end system where semantically rich information flows from perception to planning and control.

Our key innovations and their demonstrated advantages are as follows:

The Swin-Transformer-based semantic risk predictor provides a nuanced understanding of the environment, which, when leveraged by our attention-guided T-ASTAR planner, results in 30% shorter paths and 70% fewer turns compared to traditional A*.

The hierarchical architecture, where T-ASTAR provides global guidance to a TD3-based local controller, bridges the gap between static planning and dynamic execution. This synergy is critical, reducing the dynamic collision rate by 64.7% compared to decoupled approaches.

CUDA acceleration of the entire pipeline proved essential for real-world viability, achieving planning speeds up to 215 times faster and making our advanced algorithms practical for real-time decision-making.

This holistic framework represents a significant step forward from loosely coupled or modular systems, demonstrating that a deep, hierarchical integration of AI-based perception, planning, and control is crucial for achieving robust and efficient USV autonomy.

### 5.2. Future Work

Future work will focus on transitioning from simulation to real-world sea trials to address challenges like sensor noise and unforeseen environmental factors. This includes refining the energy consumption models for higher accuracy and developing predictive algorithms to handle complex, unpredictable obstacles. We also plan to explore multi-agent scenarios, coordinating several USVs to improve situational awareness and mission robustness through technologies like multi-sensor fusion.

## Figures and Tables

**Table 1:** Improved A* algorithm parameter settings.

Parameter Names	Data Description	Value
Starting point	Initial position	(0, 0)
Target point	Target position	(299, 299)
Sample size	The number of new sample points in each iteration	100
Gaussian Std	Parameters controlling the Gaussian sampling distribution	10
Initial radius	Initial search radius	150
Semi-major axis of the sampling area	Dynamically adjust the size of the sampling area based on the cost of the current path planning.	75% of the current optimal path cost
Semi-minor axis of the sampling area	Dynamically adjust the semi—minor axis of the sampling area to ensure more refined local path sampling.	25% of the distance between the starting point and the target point

**Table 2:** USV parameter list.

Parameter Names	Value	Description
∆t	0.1 s	Discrete time
m	100 kg	Quality
I	The number of new sample points in each iteration	Polar moment of inertia
L	20 m	The distance from the bow to the stern
DPV	0.01 m/s	Linear velocity sampling interval
DPW	1 rad/s	Angular velocity sampling interval
$\left[v_{min},v_{max}\right]$	[0, 1.0] m/s	Linear velocity range
$\left[\omega_{min},\omega_{max}\right]$	[−20, 20] rad/s	Angular velocity range
$C_{d}$	0.85	From Navier-Stokes simulations (Equations (5) and (6))
Re	1.2 × 10^6^	$\rho uL/\mu \left(dynamicfluidsimilarity\right)$
α	0.15	Fitted to CFD transient solutions (Equation (6))

**Table 3:** Hyperparameter settings for training the TD3 decision model.

Hyperparameter Name	Value	Data Description
The maximum number of steps per episode	1000	The maximum number of steps allowed per episode
Episodes	200	The total number of training episodes
Discount coefficient	0.95	Discount factor used to calculate future rewards
Soft update coefficient	0.005	Parameters for controlling the soft update of the target network
Policy noise	0.1	The standard deviation of the noise added to the target action
Noise clip	0.3	The range to which the noise is clipped
Strategy update delay	4	The update frequency of the policy network relative to the value network
Actor network learning rate	0.0002	Learning rate of the actor network
Critic network learning rate	0.0002	The learning rate of the critic network
Replay buffer size	2 × 10^6^	The size of the experience replay buffer

**Table 4:** Summary of Training Performance of Swin Transformer Model.

Metric	Training Set	Validation Set	Notes
Final Loss	0.085	0.123	The low loss value indicates that the model fits well
Pixel Acc, PA	98.9%	98.1%	A high PA on the validation set indicates that the model accurately determines the risk level of most pixels
Mean IoU	90.5%	88.7%	MIoU is a key indicator for semantic segmentation, and a high mIoU indicates that the model can accurately identify the boundaries of different risk areas
Epochs	150	-	
Training Time	About 5.5 h	-	On RTX 4070Ti

**Table 5:** Improved comparison results for A* algorithm with other path planning algorithms.

Algorithm	Optimal Path Length	Number of Turns	CI Value
Traditional A* ^a^	600	89	650.00
RRT ^b^	507.17	47	120.50
Bit* ^c^	460.23	26	45.30
Hybrid A* [29]	480.22	32	120.00
MPC [30]	445.13	28	15.30
FMT* [31]	430.51	28	85.25
Improved A*(ours)	425.38	27	3.31

^a^ Traditional A* implemented as described in [Hart, Nilsson & Raphael, 1968] or a standard textbook reference [10]. ^b^ RRT implemented based on A. J. La Valle, B. Sakcak 2023 [27]. ^c^ Bit* (Batch Informed Trees) implemented based on [Gammell, Srinivasa & Barfoot, 2015] [28].

**Table 6:** Analysis of the prediction error of energy consumption by ship type based on CFD simulation.

Ship Form	α Fitting Error (MAPE, %)	Root Mean Square Error (RMSE) of Energy Prediction (J)
V-shaped bow	2.3	8.7
Flat-bottomed boat	3.1	12.4

**Table 7:** CPU and GPU utilization data.

Experimental Conditions	Map Size (Grid Cells)	Reach the Target?	Is Obstacle Avoidance Successful?	CPU Usage Rate (%)	GPU Utilization Rate (%)	Remarks
Before optimization	1000 × 1000	Yes	Yes	98.2	15.4	Traditional A* algorithm, single-threaded CPU computation
	2000 × 2000	No	No	99.8	18.1	The calculation timed out, and the path planning was not completed.
After optimization	1000 × 1000	Yes	Yes	72.5	68.3	T-ASTAR + TD3 hybrid algorithm, accelerated by CUDA
	2000 × 2000	Yes	Yes	75.8	82.7	Multi-thread parallelism + GPU node expansion optimization
Ideal control group	1000 × 1000	Yes	Yes	65.0	95.0	Full GPU computing (theoretical limit)

**Table 8:** Number of collisions and successes of uncrewed vessels.

Training time	50	100	150	300	500	600	800
Number of collisions	50	95	117	218	378	471	657
The number of times you reach the finish line	0	5	33	82	122	129	143
Success rate (%)	0	0.05	0.22	0.27	0.323	0.27	0.18

**Table 9:** Experimental comparison of core planning and optimization algorithms for ablation.

Experimental Group	Configuration	Success Rate (%)	Average Path Length (m)	Average Number of Turns	Average Calculation Time (ms)	Collision Rate (%)
Group A	Traditional A* (without optimization)	62.3	600.0	89	650.0	37.7
Group B	T-ASTAR (without optimization)	78.5	460.2	26	45.3	21.5
Group C	TD3 Learning from Scratch	68.9	507.2	47	120.5	31.1
Group D	T-ASTAR + TD3	92.4	425.4	27	3.3	7.6

**Table 10:** Comparison of Perceptual Module Ablation Experiments.

Experimental Group	Perception Mode	Success Rate (%)	Average Path Length (m)	Average Number of Turns	Average Calculation Time (ms)	Collision Rate (%)	Cumulative Time (s)/Number of Times Entering High-Risk Areas
Group E	Simple perception (color segmentation + edge detection)	65.2	550.6	38	120.0	34.8	42.5/18
Group F	Swin-Transformer (this paper)	88.7	435.8	24	85.3	11.3	8.2/3
Group G	Ideal perception (global perfect map)	98.5	412.4	18	70.5	1.5	0.0/0

**Table 11:** T-ASTAR without Attention Mechanism (Group H). (↑ indicates an increase).

Metric	Full T-ASTAR	w/o Attention	Change
Node Expansions	2148 ± 217	3034 ± 301	↑ 41.3%
Planning Time (ms)	45.3 ± 4.2	63.7 ± 5.8	↑ 40.6%
Path Curvature (rad/m)	0.75 ± 0.08	0.92 ± 0.11	↑ 22.7%
Turns > 45°	4.2 ± 0.9	7.1 ± 1.3	↑ 69.0%

**Table 12:** TD3 without Energy Reward (Group I). (↑ indicates an increase).

Metric	Full TD3	w/o Energy Reward	Change
Energy Consumption (kJ)	115.8 ± 6.2	143.2 ± 8.9	↑ 23.7%
Thrust Variance ($\tau_{u}$)	0.081 ± 0.011	0.127 ± 0.018	↑ 56.8%
Max Yaw Rate (rad/s)	1.05 ± 0.13	1.62 ± 0.21	↑ 54.3%
Wave Drag Power (W)	420 ± 38	583 ± 52	↑ 38.8%

**Table 13:** Test Groups.

Group	Configuration	Key Difference
A	T-ASTAR + PID Tracker	No TD3; rigid path tracking
B	T-ASTAR + TD3 (No Feedback)	TD3 tracks path but no online feedback to T-ASTAR
C (Full Ours)	T-ASTAR + TD3 (w/Feedback)	Execution data fine-tunes T-ASTAR’s cost predictor (Equation (3))
D	Pure TD3 (No Global Path)	Learns from scratch without T-ASTAR guidance

**Table 14:** Results about group A to D. (↑ indicates an increase, ↓indicates a decline).

Group	Success ↑	Collisions ↓	Adaptation Gain ↑	Path Dev. ↓	Replan ↓
A: PID Tracker	71.2	28.8	0.0	12.4	0
B: TD3 (No FB)	85.6	14.4	18.3	5.1	6.2
C: Full	92.4	7.6	41.7	2.1	1.8
D: Pure TD3	68.9	31.1	22.5	N/A	N/A

**Table 15:** Test Groups.

Group	Perception Input	Planner
**E**	Binary Obstacle Map	Standard A*
**F**	Binary Obstacle Map	T-ASTAR
**G**	Swin-T Risk Map	Standard A*
**H (Full Ours)**	Swin-T Risk Map	T-ASTAR

**Table 16:** Results about group E to H. (↑ indicates an increase, ↓ indicates a decline).

Group	Success ↑	Collisions ↓	High-Risk Exp. ↓	False Obst. ↓	Semantic Util. ↑
E: Binary + A*	62.3	37.7	38.2	16.4	51.2
F: Binary + TA	70.1	29.9	32.7	16.4	63.8
G: Swin + A*	76.5	23.5	18.9	5.3	72.1
H: Full	88.7	11.3	8.2	5.3	89.6

## Data Availability

The original contributions presented in this study are included in the article.
